# Molecular Modeling Is Key to Understanding Supramolecular Resorcinarenyl Capsules, Inclusion Complex Formation and Organic Reactions in Nanoconfined Space

**DOI:** 10.3390/molecules30122549

**Published:** 2025-06-11

**Authors:** Maxime Steinmetz, David Sémeril

**Affiliations:** Synthèse Organométallique et Catalyse, UMR-CNRS 7177, Institut de Chimie de Strasbourg, Strasbourg University, 67008 Strasbourg, France; maxime.steinmetz@etu.unistra.fr

**Keywords:** in silico techniques, self-assembly capsule, resorcin[4]arene, host–guest chemistry, supramolecular interaction

## Abstract

This review highlights how advances in silico techniques have shed new light on phenomena in confined supramolecular resorcinarene-based systems. Computational studies have provided detailed insights into capsule formation, their dynamic behavior, guest encapsulation and reaction mechanisms within these hosts, often revealing information that experimental methods cannot reach. The focus is placed on the self-assembly of resorcin[4]arenes, pyrogallol[4]arenes, velcrands, and octa acid systems. These computational studies complement experimental findings and, in many cases, offer new perspectives that are inaccessible using experimental techniques alone. Supramolecular architectures are growing in complexity the role of in silico approaches is becoming indispensable. They offer a way to design rationally and understand host–guest chemistry more deeply.

## 1. Introduction

The rapid development of in silico techniques over the past few decades has completely changed our approach to chemical research [1,2,3,4,5]. Initially limited by computational power and model sophistication, these methods now allow the exploration of increasingly complex supramolecular systems. Molecular modeling has been a particular boon to supramolecular chemistry [6,7,8,9,10,11,12]. Modern analytical techniques have greatly improved the characterization of supramolecular assemblies, but they still exhibit intrinsic limitations, particularly in terms of spatial and temporal resolution at the atomic scale. These limitations hinder our understanding of the dynamic and structural aspects of certain systems, such as molecular capsules.

In silico studies are now essential complementary tools. These simulations decisively address certain experimental limitations and provide additional insights into the structure, dynamics, and molecular interactions within supramolecular assemblies. They undoubtedly contribute to a more integrated and comprehensive understanding of the properties of these systems by enriching the experimental data.

The chemistry of supramolecular containers is a significant sub-discipline of supramolecular chemistry. Various strategies have been developed, leveraging non-covalent interactions such as ionic interaction, coordination bonds, hydrogen bonds, or halogen bonds [13]. Assemblies based on hydrogen bonding, in particular, have attracted particular attention due to their dynamic nature [14]. Resorcin[4]arenes are one of the most utilized macrocycles in this field. They serve as versatile molecular platforms [15,16]. These macromolecules are known to self-assemble in organic solutions in the presence of water, as demonstrated by Atwood [17]. Their remarkable chemical modularity has expanded the range of possible supramolecular architectures, generating sustained interest in the scientific community, particularly in the field of catalysis [18,19,20,21,22].

**Figure 1:**
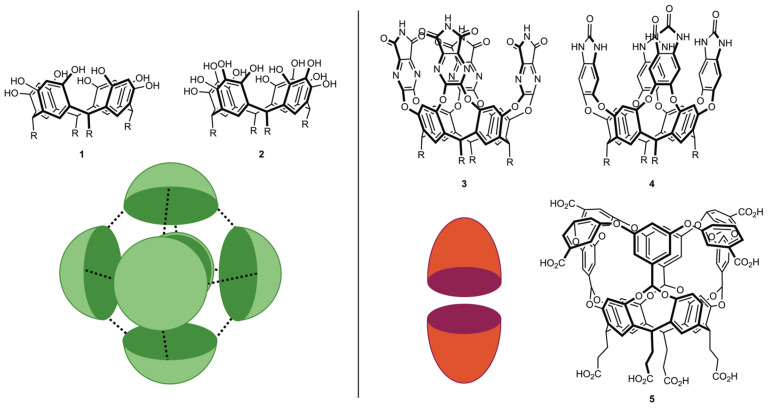
Resorcin[4]arene (**1**) and pyrogallol[4]arene (**2**) forming a hexameric capsule (**left**) and velcrands (**3** and **4**) and octa acid (**5**) forming dimeric assemblies (**right**).

In this review, we will explore the contributions of in silico approaches to understanding these supramolecular assemblies. We highlight the methodologies and computational levels employed. The systems studied include resorcin[4]arenes **1** self-assembled into hexameric capsules (Atwood’s capsule), their pyrogallol[4]arene counterparts **2**, velcrands **3** and **4**, and, finally, octa acid-based **5** assemblies (Figure 1). The four sections will demonstrate how molecular simulations complement experimental data and provide unique perspectives on the dynamics and properties of these fascinating assemblies.

## 2. Discussion

### 2.1. Atwood Capsule

**Figure 2:**
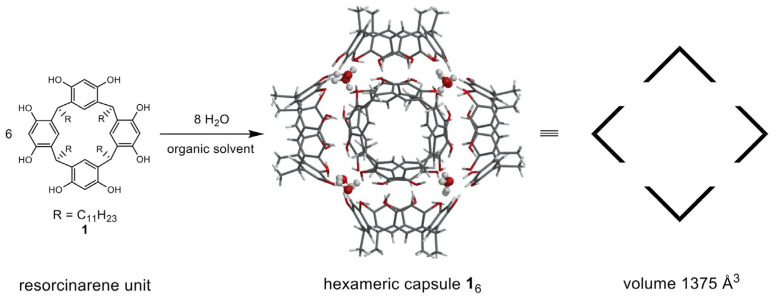
Formation of the Atwood capsule in an apolar solvent in presence of water.

The self-assembly of resorcin[4]arene **1** in the presence of water in organic media [23,24,25], discovered by Atwood and MacGillivray in 1997 [17], represents a significant advance in supramolecular chemistry. This self-assembly process, which involves the formation of a capsule from six resorcin[4]arene **1** molecules and eight water molecules in non-polar solvents (Figure 2), offers numerous advantages. The resulting cavity is hydrophobic; its diameter and volume were determined from the X-ray structure to be 24.3 Å and 1375 Å^3^, respectively, making it an interesting structure for the encapsulation of various compounds. Indeed, the confined space of the capsule allows efficient encapsulation of a wide variety of organic compounds [26,27,28,29,30], as well as metal complexes [31,32,33,34,35,36,37,38,39,40,41,42,43]. Some of these encapsulated systems can be used for catalysis, showing marked differences in reactivity and selectivity compared to free catalytic systems [40,41,42,43,44,45,46]. These differences, sometimes subtle, have motivated in-depth studies to understand the effects of supramolecular confinement in this type of capsule.

This section will be divided into three subsections. The first will include studies on the intrinsic properties of the resorcin[4]arene capsule, examining its formation and the role of water. The second will focus on work related to the encapsulation of different species within the capsule, analyzing what leads to the grafting of different species. Finally, the third subsection will deal with mechanistic studies, focusing on the impact of supramolecular confinement on the catalytic processes and molecular dynamics of the system.

#### 2.1.1. Capsule Assembly

- Early-stage formation of the hexameric capsule.

The groups of Capelli and Piccini have studied in silico the preliminary stages of the resorcinarenyl capsule formation [47]. Given the rapidity of the self-assembly process, current experimental techniques do not allow for direct observation of the dynamics; recourse to simulation allows us to overcome this problem.

The formation of the capsule **1**_6_ involves a series of slowly activated processes, characterized by significantly large free energy barriers between structural intermediates [48]. Because of these barriers, classical molecular dynamics is not suitable for capturing the transitions between states, which led the authors to employ metadynamics [49,50], a simulation technique more suitable for studying the various intermediates. Simulations were performed in chloroform with eight equivalents of water per capsule. All their calculations were performed using GROMACS 2019.223 patched with PLUMED 2.6 [51,52].

The results show that dimer **1**_2_ and hexamer **1**_6_ structures are thermodynamically favored over the monomer **1**. In the second step, the team established the free energy surface for the assembly process, which allowed them to map the structural intermediates between each metastable state. Their analysis revealed several intermediate states: the first metastable state is an association of dimers (**1**_2_)_n_ interacting in pairs (forming aggregates of n = 2 or 3 dimers), while the formation of isolated monomers or dimers is not located in a stable free-energy region. The second metastable state corresponds to a tetramer interacting with a dimer (**1**_4_•**1**_2_). Although relatively stable, this assembly is less stable than dimers or the final hexamer. The final state of the assembly is the hexamer **1**_6_, the most stable and predominant structure in this equilibrium. The team observed that the equilibrium is mainly between dimeric (**1**_2_)_n_ and hexameric species **1**_6_. Furthermore, the authors note that water does not have a limiting kinetic effect due to its rapid diffusion compared to that of the resorcinarene monomers.

In their work, the group also examined the average number of solvent molecules encapsulated within the hexameric capsule. NMR analysis in chloroform suggests the presence of six [53] to eight [46] chloroform molecules within the structure. However, this method seems to reveal only those molecules that interact directly with the resorcinarene subunits. In contrast, computational simulations determined an average number of encapsulated solvents of eight, a result that differs from the experimental observations. This difference suggests that the NMR studies may underestimate the actual number of chloroform molecules encapsulated in the capsule by highlighting only molecules closely associated with the resorcinarene subunits, whereas the simulations include all the solvents present.

- Water molecules.

An essential parameter for the self-assembly of the capsule is the ratio of water to the capsule itself. Capsule formation does not occur in the presence of anhydrous chloroform [25,54]. The effect of the water concentration is even more important to study because the mechanisms of encapsulation and release of guests within this capsule are not fully understood. Some publications suggest the existence of pentameric intermediates **1**_5_ for encapsulation and during certain catalytic reactions, as well as the possibility of partial opening of the capsule to facilitate guest exchange [45,55]. In addition, a study by Tiefenbacher and co-workers observed that the catalytic activity of the capsule varies with water concentration [56].

**Figure 3:**
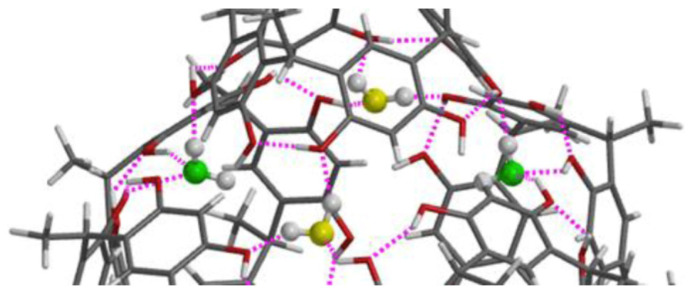
Description of the hydrogen bonds (in magenta) forming the hexameric capsule in organic solvents in the presence of water. The two types of water are represented in yellow and green.

X-ray crystallographic analysis revealed that the eight structural water molecules within the assembly can be categorized into two distinct groups. Four of them act as double hydrogen bond donors and single acceptors (Figure 3, oxygen atoms in yellow), whereas the remaining four function as single donors and double acceptors (Figure 3, oxygen atoms in green). The latter group exhibits a hydrogen atom oriented toward the interior of the capsule, enabling potential interactions with encapsulated guest species. The capsule exhibits acidic behavior, with a global pKa value of 5.9 [57]. In silico pKa estimations for each water molecule were performed using Gaussian 09 at the PM6 level of theory [58,59,60]. These calculations revealed significant variations in the acidity of the individual water molecules, particularly for the second group, which displays microenvironment-dependent pKa values around 2.5. It is important to understand that the water molecules observed in the solid-state structure are in dynamic equilibrium with bulk solvent molecules in solution.

To learn more about the role of water, several studies, both experimental and in silico, have been conducted to understand the behavior of the capsule as a function of the relative amount of water. Thompson and co-workers studied the water diffusion coefficient as a function of the water/capsule ratio [61,62], for which they performed molecular dynamics simulations using the Large-Scale Atomic/Molecular Massively Parallel Simulator (LAMMPS) [63,64] software. The data obtained were then compared with the experimental results of the group of Cohen [24]. From an experimental point of view, a rapid exchange between different water populations on the NMR time scale was demonstrated. Indeed, a single water peak was observed in the ^1^H NMR spectra for all water/capsule ratios. This leads to a diffusion coefficient measured by NMR, which is a weighted average of the diffusion coefficients of the different water populations, including molecules interacting with the assembly and those remaining free in solution. As far as diffusion coefficients are concerned, that of the capsule remains practically invariant as a function of the water/capsule ratio, unlike that of water, which shows a notable increase, followed by a plateau as the ratio increases. This observation allowed them to model the behavior of the water diffusion coefficient as a simple adsorption isotherm, providing an excellent correlation between experimental results and simulations. This model highlights a dynamic equilibrium between the so-called structural water molecules, which are integrated into the hydrogen bond network that holds them together, and those that remain free in the solution. However, in order to more accurately describe the experimental data, it was necessary to introduce an additional category of water molecules, called “bound”. The presence of encapsulated water molecules was also observed in their simulations. The calculations performed showed that, on average, 6.3 water molecules are encapsulated or attached in the capsule structure, in addition to the eight structural water molecules. This value is in agreement with the value of six molecules obtained from NMR measurements.

To study the behavior of attached or encapsulated water molecules, the authors examined the probability of a water molecule’s presence as a function of its distance from the capsule’s center of mass and as a function of the relative water concentration. In all simulations, the eight structural water molecules were always present, regardless of the water concentration. The second most stable type of water observed is encapsulated water. The presence of these encapsulated water molecules in chloroform had already been suggested experimentally [53]. In fact, encapsulated water molecules were observed at a ratio of 6/14 (resorcinarene/water). Beyond this ratio, the number of encapsulated water molecules reaches a plateau at 2–2.5 molecules per capsule, from a ratio of 6/16, despite the continuous increase in water concentration. The authors also observed the attachment of water molecules to the outside of the capsule. These molecules are associated with structural water molecules, forming bound water clusters in a variety of arrangements. These bound water molecules are detected at a ratio of 6/16. However, they only become significantly present at higher ratios (6/40 to 6/50), where the concentration reaches an average value of about one bound water molecule per capsule. However, the number of bound water molecules can reach up to 17 molecules per bound cluster. The team also studied the free energy profiles of each type of water molecule as a function of the relative water concentration. They found that, as this concentration increased, the free energy for each category of water molecule became progressively more favorable. Furthermore, a van’t Hoff analysis showed that the free energy surface is mainly influenced by entropic contributions. The exchange kinetics between the different water populations have also been studied in detail by the group of Thompson. The transition from encapsulated or bound water molecules to structural water molecules is fast, on the order of 5 to 10 ps. In contrast, the reverse exchange, i.e., from structural water molecules to encapsulated or bound forms, is much slower, with times on the order of 100 ps to a few ns. The slowest dynamics concern the transfer of free water molecules to bound molecules, a process that takes between 3 and 15 ns, depending on the relative water concentration. Exchanges between these populations are fast on the NMR timescale, as observed experimentally [61,62].

**Figure 4:**
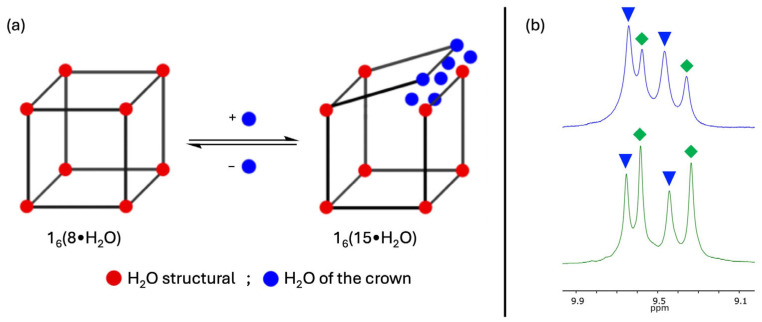
(**a**) Representation of the incorporation of water molecules into **1**_6_ leading to **1**_6_ (15•H_2_O); (**b**) ^1^H NMR spectrum of phenols **1**_6_(8•H_2_O) (green square) and **1**_6_(15•H_2_O) (blue triangle) at different H_2_O concentrations (CDCl_3_ saturated in H_2_O at the **bottom** and after addition of H_2_O at the **top**).

A classical molecular dynamics study by Reek and co-workers, performed with pmemd.CUDA [64,65], suggests the formation of a water crown around one of the six resorcinarene units, forming the capsule **1**_6_(15•H_2_O) [66]. In their simulations, the presence of more than 14 water molecules per capsule enables the formation of this crown, which is positioned at the top of one of the resorcinarene units (Figure 4a). At least six additional water molecules are required to initiate this extension. These molecules are more mobile than the structural molecules and are not strongly localized. Although the formation of a water crown is thermodynamically favored, the formation of multiple crowns around the same capsule is energetically unfavorable because it leads to too much entropic loss. These results were supported by NMR investigations, including both ^1^H NMR and DOSY experiments. In particular, the splitting observed in the phenolic proton signals in the ^1^H NMR spectrum was unambiguously attributed to the presence of both capsule **1**_6_(8•H_2_O) and **1**_6_ (15•H_2_O) in equilibrium, rather than to the intrinsic chirality of the assembly, as had previously been assumed (Figure 4b). They also showed that the formation of the water crown induces an increase in capsule acidity and reactivity, for example, in the Diels–Alder reaction.

It is possible to partially replace the structural water molecules with either alcohols or halogens [24,65,66,67,68,69,70]. In the latter case, Szumna and co-workers demonstrated that the eight structural water molecules present in the capsule could be replaced by an equimolar number of halides while maintaining the hexameric assembly [71]. It has been shown that this capsule can be obtained in solvents such as benzene or tetrahydrofuran, which are weak solvents for anions. However, these assemblies are not observed in chloroform. The formation of this hexamer has been supported by both experimental and DFT (B3LYP functional, employing the 6-31g basis set) [72,73] studies using the Gaussian 09 program suite [59]. These calculations have allowed the electrostatic potential of resorcinarene to be plotted, highlighting broad regions of positive electrostatic potential along the phenolic groups and upper rim hydrogens. These regions are expected to favor interactions with anions. Thus, three hydrogen bonds are formed for each halide, with bond distances varying between 3.1 and 3.3 Å for chlorides. The distances between chlorides were calculated to be 6.2 Å, indicating the absence of significant repulsive interactions between these anions. The formation of the capsule was also supported by several experimental studies.

#### 2.1.2. Formation of Inclusion Complexes

Given the substantial size of the cavity, 1375 Å^3^, it is plausible that guest molecules can adopt different conformations within it and that dynamic exchange between these different configurations can occur. Although some of these phenomena can be addressed by experimental studies, it should be noted that not all systems can be fully characterized by this approach due to the rapidity of the exchange and the inherent limitations of the techniques. For a deeper understanding of these dynamics, in silico studies provide a valuable alternative.

A well-known emblematic example of guests for this capsule is tetra-alkyl ammoniums [26]. It is important to keep in mind that the encapsulation of a guest inside the hexameric capsule requires some energy, corresponding to the energy needed to open a door in the capsule (energy needed to break down hydrogen bonds) as well as the energy required to push guests through this door. Using DFT at the PBE-D3/def2-SVP/Ɛ level with TURBOMOLE v. 5.5–5.6 [74,75,76,77,78]. Tiefenbacher and co-workers have elucidated the reasons for the efficient encapsulation of these ammoniums [79]. Their work showed that the hexameric capsule promotes stabilization of the cations through π•••cation interactions, as would be expected. But that is not all: the resorcinarenyl capsule is also able to stabilize the counter anion. To demonstrate this, the authors calculated the electrostatic potential map of the inner surface of the capsule. The results indicate that the hydrogen atom on the top surface of resorcinarene, as well as the protons of the structured water molecules, are positively charged (similar to the capsule maintained by halide previously described). These positively charged sites can then interact with the anion, helping to stabilize it within the capsule. This mechanism reduces the energy associated with charge dissociation compared to a situation where the anion would not be part of the assembly, thus facilitating the stability of the whole.

To go further, the group of Rebek performed molecular mechanics calculations on the hosted tetra-alkyl ammonium salts to better understand the specific chemical shifts observed in the ^1^H NMR spectrum for the proton of the alkyl chains [55]. In this example, in silico studies using the Wab Lab Viewer program [80] were performed on three different ammonium salts, namely tetra-hexyl ammonium bromide (**6**), tetra-heptyl ammonium bromide (**7**) and tetra-octyl ammonium bromide (**8**). Results from ^1^H NMR spectroscopy and molecular mechanics calculations show that encapsulation occurs without refolding of the hexyl chains. Conversely, for ammonium salts with longer alkyl chains (heptyl **7** and octyl **8**), the size of the ammonium salts becomes too large to allow their encapsulation with a linear conformation of the alkyl chain. A folding of the alkyl chain occurs to accommodate the limited space provided by the capsule. This phenomenon was found to occur at the fourth carbon atom of the alkyl chain. The group of Shivanyuk reported the co-encapsulation of a calix[4]arene **9** and a tetra-methylammonium iodide (**10**) inside the resorcinarenyl capsule [81]. Molecular mechanics calculations were carried out based on crystallographic coordinates obtained by MacGillivray and Atwood [17] and calculated with MMX force [82]. This simulation predicted that the calixarene is encapsulated “face-to-face” with respect to a unit of the hexamer. This prediction is corroborated by the shielding effect observed in NMR spectroscopy (^1^H and NOESY), which supports the proposed configuration. The energetically optimized structure shows that ammonium can indeed be encapsulated in this arrangement. Two positions corresponding to energy minima were identified, and NMR analysis revealed that most of the ammonium **10** is located in the calixarene cavity.

**Figure 5:**
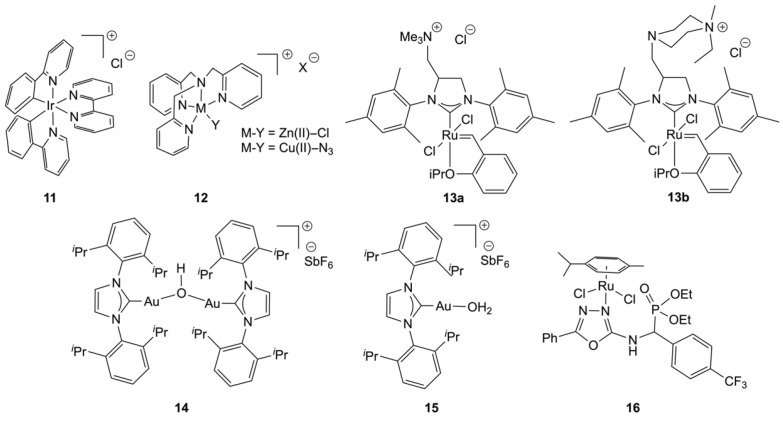
Selected organometallic complexes **11**–**16** studied by in silico inside the Atwood capsule.

The self-assembling capsule can also host organometallic complexes [31,32,33,34,35,36,37,38,39,40,41,42,43]. A number of the latter inclusion complexes have been studied in silico by molecular methods (Figure 5) [37,38,39,40,41,83,84].

**Figure 6:**
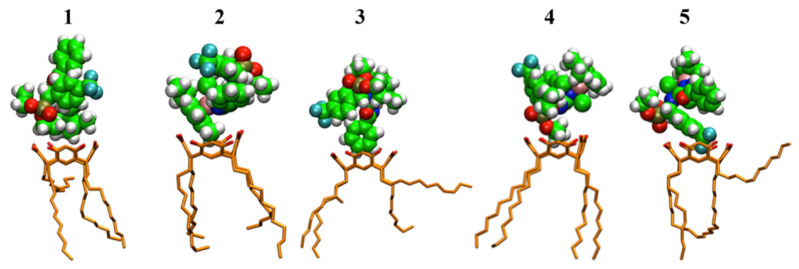
Selected orthogonal views of interactions between a resorcinarene unit and the ruthenium complex **16**: (1) *p*-cymene π-system; (2) aromatic protons of the *p*-cymene ring; (3) phenyl ring attached to the oxadiazole; (4) CH protons of the P(O)(OEt)_2_ groups; (5) CF_3_ substituent.

The group of Umakoshi studied the modeling of the inclusion complex formed by the cationic iridium complex [Ir(ppy)_2_(bpy)]X (**11**, Figure 5) (ppy = 2-phenylpyridinate and bpy = 2,2′-bipyridine) in the hexameric capsule. The geometries of the resorcinol capsule and the guest **11** were based on their individual X-ray structures. The simulations have clearly shown that the hexameric capsule has a cavity large enough to trap the iridium complex cation. In addition, the VOIDOO program estimates that the remaining space in the capsule is of the order of 40–70 Å^3^, which is sufficient to allow also the encapsulation of the small cation (chloride, bromide, iodide, nitrite, nitrate or tetrafluoroborate) and to form an ion-pair in the inner space of the capsule, leading to a large blue shift in the emission wavelength of the iridium complex **11** [83]. Reinaud, Colasson and co-workers also observed that the triflate counteranion, unlike BArF^−^ (BArF^−^ = tetrakis(3,5-bis(trifluoromethyl)phenyl)borate), can also be accommodated in the cavity with the organometallic cation [ZnCl(TMPA)]^+^ (TMPA = tris(2-pyridylmethyl)amine) (**12**, Figure 5) [37]. The authors also performed molecular modeling by docking the zinc cation with LigandFit [85]. For the [ZnCl(TMPA)]^+^ complex, the calculations revealed two preferred binding orientations. Several CH•••π interactions and CH_2_•••OH_2_ interactions were observed in the simulations. Furthermore, the encapsulation of three chloroform molecules was observed, which is consistent with a favorable packing coefficient [86]. These results are consistent with the cleavage patterns observed in the NMR spectra. In addition, the in silico studies showed that the metal-bound chloride ion does not form hydrogen bonds with the OH groups of the capsule in the simulation. In contrast, the [CuN_3_(TMPA)]^+^ complex, which can be used as a probe for hydrogen bonding by IR spectroscopy [87,88], revealed the formation of relatively strong hydrogen bonds with the azide during encapsulation. The simulation of this complex showed two conformations similar to those observed for the zinc complex. In one of these conformations, a hydrogen bond was observed between the azide and the hexameric capsule, with a bond length of 1.52 Å, which is consistent with the strength of the hydrogen bond as measured by IR spectroscopy. On the other hand, the groups of Fogg and Reek investigated two modified Grubbs complexes (**13a** and **13b**, Figure 5) by molecular dynamics using the CUDA-enabled Amber14 software suite [41]. They first demonstrated that this complex could indeed fit into the interior of the capsule. They then simulated the diffusion coefficient of the complex and obtained a value in excellent agreement with the experimental data (logD(simulated) = −9.57 vs. logD(measured) = −9.62) [24]. They also observed a small expansion of the assembly due to the encapsulation of the guest, to approximately 1930 Å^3^, as determined by the Conolly roll method [89]. For their part, Echavarren, Ballester and co-workers carried out a detailed study of encapsulation behavior of gold-based complexes, employing molecular modeling with the MM3 force field [90]. According to the authors and based on molecular simulations, when the gold precursors themselves cannot be directly encapsulated, the addition of water facilitates the formation of *μ*-OH pseudo-dimer complexes (**14**, Figure 5) [91], which could dissociate into cationic gold complexes bearing an H_2_O ligand (**15**, Figure 5), the latter species being easily encapsulated [39]. In this context, we investigated the encapsulation of a neutral ruthenium-based complex in the hexameric host through molecular dynamics simulations using the AMBER.18 GPU software [92] (**16**, Figure 5) [40]. As observed experimentally by ^31^P and ^19^F NMR spectroscopy performed on the inclusion complex, the simulation showed that the complex is in rapid motion within the cavity whose volume remains relatively stable (2367 Å^3^ versus 2324 Å^3^ for the simulated “empty” capsule). The theoretical study revealed that the ruthenium complex strongly interacts with the inner walls of the capsule through supramolecular interactions of π•••π, CH•••π and CF•••π types, as observed in Figure 6.

#### 2.1.3. Study of Mechanisms Inside the Atwood Capsule

**Scheme 1:**
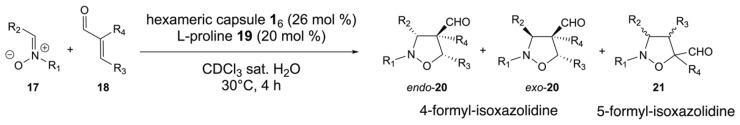
1,3-dipolar cycloaddition of nitrones with *α*,*β*-unsaturated aldehydes catalyzed with L-proline inside the hexameric capsule.

The group of Neri was interested in exploiting the acidic properties of water molecules of the hexameric capsule architecture in organic reactions. In order to better understand the role played by the water molecules and the inner walls of the cavity, in silico studies were carried out. This research group investigated the 1,3-dipolar cycloaddition between a nitrone **17** and an *α*,*β*-unsaturated aldehyde **18** catalyzed by L-proline **19** (Scheme 1) [44]. In the absence of the capsule, no conversion was observed. Conversely, in the presence of the capsule, important conversions, substantial regioisomeric 4-formyl-isoxazolidine/5-formyl-isoxazolidine **20** ratios (up to 100/0) with high diastereoisomeric (*endo*/*exo*) ratios (up to 96/4) and enantiomeric excess (up to 95%, (4*R*)-product) were observed, except when acrylaldehyde was employed as a substrate.

**Scheme 2:**
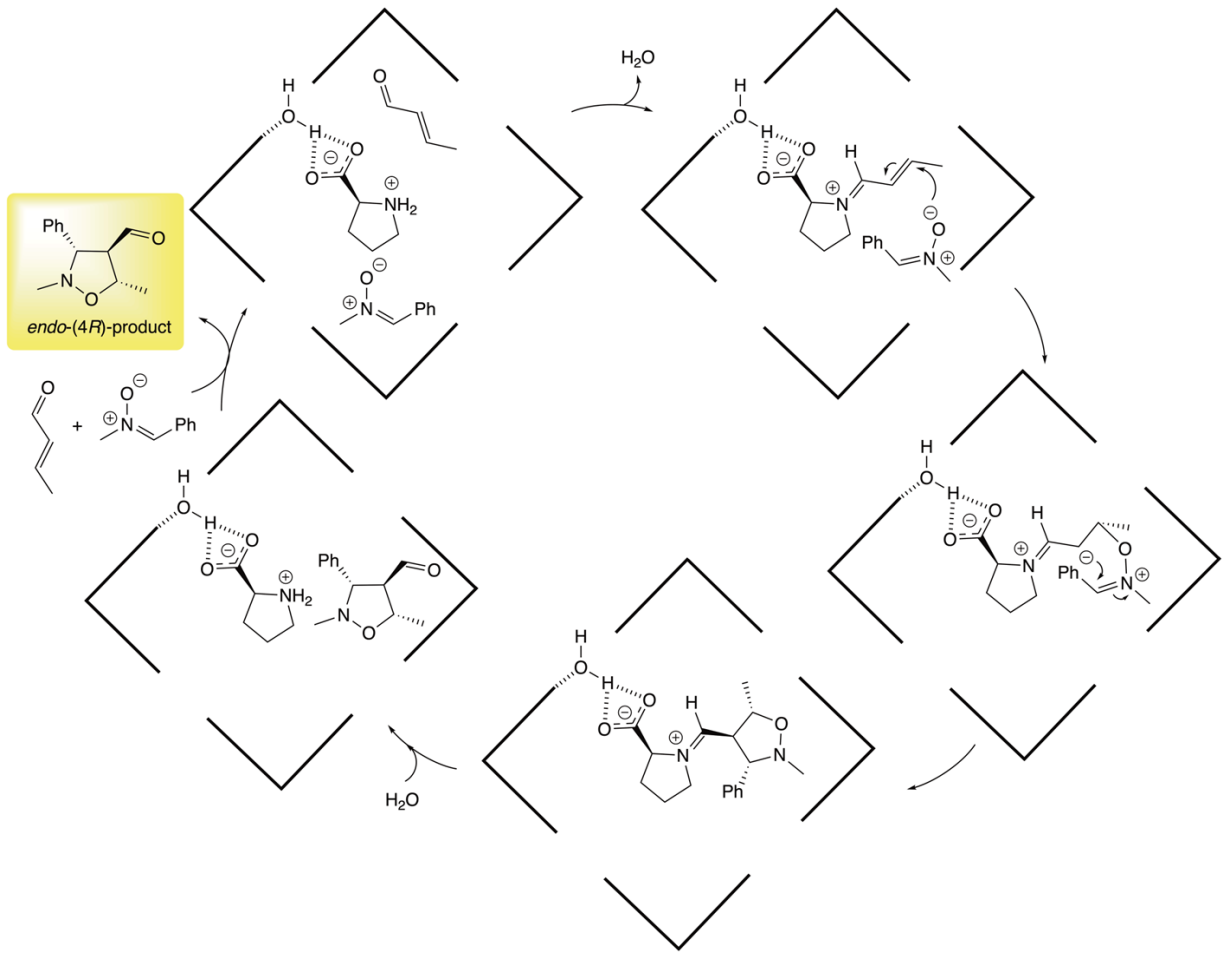
Proposed mechanism for the *meta*-channel as the least energetic pathway.

To explain these catalytic results, quantum chemical investigations were performed using *N*-methyl-C-phenyl nitrone **17a** and (*E*)-crotonaldehyde **18a** as substrates. The simulations were carried out with the Gaussian 09 program using the PM6 semiempirical Hamiltonian [59,60] and showed that the encapsulation of the reactants occurs in the following order: L-proline **19**, the nitrone **17a**, and then the aldehyde **18a**. Hydrogen bonds were observed between the oxygen atoms of the nitrone **17a**, aldehyde **18a**, or carboxyl groups and the structural water molecules, explaining the observed stabilization. The iminium intermediate, resulting from the addition of L-proline and the aldehyde, was formed within the capsule and served as a catalyst for the 1,3-dipolar cycloaddition of the nitrone. Once the product is formed, hydrolysis can take place either inside or outside the capsule. They show that the latter case appears to be the least favorable, but if the entry of the nitrone **17a** is considered along with the expulsion process, the energy cost is reduced, making the catalytic cycle viable. A total of eight possible products and, correspondingly, eight reactive channels were identified. The study of the free energy profiles showed that the *meta*-channel is less energetic, which explains the observed regioselectivity in favor of the *endo*-(4*R*)-product **20a** (Scheme 2). It is noteworthy that the hexameric capsule has demonstrated a degree of flexibility during the process of inclusion complex formation [40]. This capacity for accommodation to the host should be sustained through the utilization of reaction intermediates during catalytic processes. This adaptability of the capsule should allow, for example, better stabilization of cationic intermediates by the inner aromatic walls of the capsule.

**Scheme 3:**
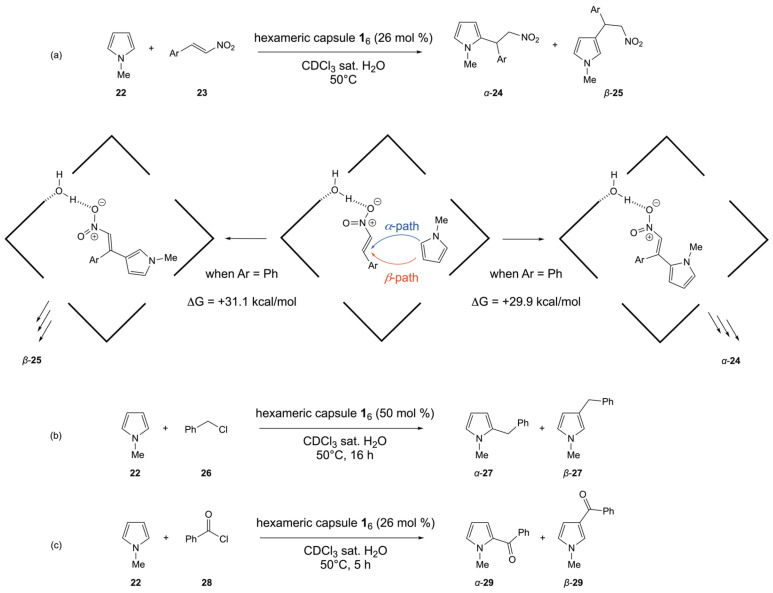
Michael-type Friedel–Crafts alkylation between *N*-methylpyrrole **22** and (*E*)-(2-nitrovinyl)arene **23** (equation (**a**) with two Wheland intermediates leading to the two regioisomers), benzylic chloride **26** (equation (**b**)) and benzoyl chloride **28** (equation (**c**)).

Similarly, the authors studied the Michael-type Friedel–Crafts alkylation between *N*-methylpyrrole **22** and (*E*)-(2-nitrovinyl)arene **23** (Scheme 3a) [93]. As previously observed, no conversion was measured in the absence of the hexameric capsule. Under optimal reaction conditions, in the presence of the supramolecular assembly and at 50 °C, quasi-full conversions were obtained, except when sterically hindered (*E*)-(2-nitrovinyl)arene was employed ((*E*)-1-methoxy-2-(2-nitrovinyl)benzene **23a** and (*E*)-1-chloro-2-(2-nitrovinyl)benzene **23b** with conversions of 70 and 80%, respectively). A marked regioselectivity for the *α*-products, up to 85%, was obtained. In order to better understand these observations, quantum mechanical investigations were performed using the ONIOM method included in the Gaussian 16 package [94]. Since two regioisomers are possible, the mechanistic analysis helped to explain the observed regioselectivity in the presence of the capsule. Calculations showed that (*E*)-(2-nitrovinyl)arene **23** was the first to host the capsule, followed by *N*-methylpyrrole **22**. Hydrogen bonding with the structural water molecules of the capsule occurs with the nitro group, which facilitates this step. The calculated enthalpy was negative, although this was accompanied by an entropic penalty. It is well established that the formation of the two regioisomers depends on two different pathways leading to two different Wheland intermediates. In this Michael–Friedel–Crafts-type mechanism, the pathway leading to the *β*-product **25** is higher in energy (*δ*(ΔG) of 1.2 kcal/mol), which explains the preferential formation of the *α*-regioisomer **24**, in agreement with the experimental data. The in silico study highlights that the capsule plays a key role in activating the substrates and stabilizing the reaction intermediates through hydrogen bonding and π•••cation interactions. Furthermore, no inhibition by the capsule is observed, as the release of the product is favored over the encapsulation of the reactants. In addition, the theoretical study demonstrates that the capsule effectively controls the reactivity by protecting the nitronic acid intermediate from common degradation pathways observed in the bulk medium.

**Scheme 4:**
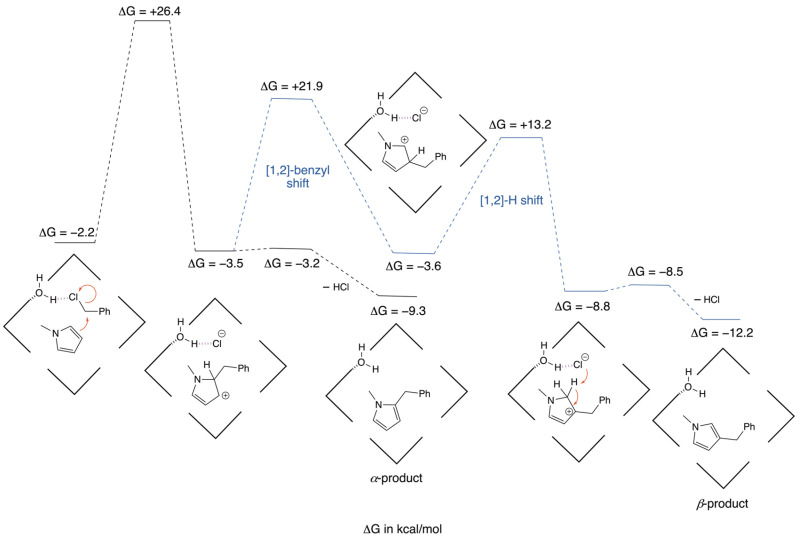
Gibbs free energy profile for the Friedel–Crafts alkylation of *N*-methylpyrrole with benzyl chloride inside the resorcinarenyl capsule.

Neri and co-workers investigated in detail the carbon-chloride bond activation by the capsule. In the supramolecular assembly, the presence of a hydrogen bond formed between one of the structural water molecules and the chlorine atom of the substrate makes the C-Cl bond weaker and consequently the species more reactive. The first example investigated was the Friedel–Crafts alkylation of aromatic substrates with benzylic halides (Scheme 3b) [58]. The investigations demonstrated that in the absence of the hexameric capsule at 50 °C in CDCl_3_ saturated with water, no reaction occurred after 16 h. Repeating the run in the presence of the resorcinarenyl capsule (50 mol %) led to high conversions of *N*-methylpyrrole **22**; the conversions depend drastically on the benzylic halides and increased in the order I (no conversion) << Br (72%) ≈ F (75%) < Cl (81%). The main formed product, the *β*-product **27**, adopts an unusual regiospecificity for this reaction with an *α*-/*β*-products ratio of up to 6/94 [95,96,97,98]. To explain this out-of-the-ordinary regioselectivity, quantum chemical investigations using the ONIOM method incorporated in the Gaussian 09 package were carried out on the model reaction *N*-methylpyrrole **22** and benzyl chloride **26** [59]. The calculations showed that the Friedel–Crafts alkylation takes place inside the hexameric capsule. Thanks to a hydrogen bond between a water molecule of the supramolecular structure and the chlorine atom of the substrate **26** (distance Cl•••H of 2.34 Å), the formation of the hetero-binary inclusion complex between the two substrates and the capsule has a slightly negative enthalpy. In addition, the calculations indicate that the *α*-product **27** is formed preferentially via a Wheland intermediate at ΔG = 26.4 kcal/mol. It has also been calculated that the retro Friedel–Crafts reaction requires a modest energy barrier of 6.1 kcal/mol, which makes the reaction of the product reversible. Therefore, the [1,2]-benzyl shift can occur via a transition state at ΔG = 21.9 kcal/mol. Then, the formed intermediate can rearrange itself via a [1,2]-H shift to generate the Wheland intermediate at ΔG = 13.2 kcal/mol. Finally, the *β*-product **27** is generated by rearomatization. The calculated enthalpy for the generated inclusion complex made of the *β*-product and the hexameric capsule (ΔG = −12.2 kcal/mol) is thermodynamically more stable than the inclusion complex made with the *α*-product **27** (ΔG = −9.3 kcal/mol) and explains the unusual observed regioselectivity of the Friedel–Crafts alkylation inside the supramolecular capsule (Scheme 4).

It is interesting to note that in the case of the Friedel–Crafts alkylation of *N*-methylpyrrole **22** with benzoyl chloride **28**, calculations demonstrated that the retro Friedel–Crafts reaction was very slow, due to a significant activation barrier of ΔG = 33.6 kcal/mol for the *α*-product **29**; although the thermodynamic product is the *β*-product **29**, the formation of both products was observed with an *α*-/β-products ratio of 40/60 (Scheme 3c) [99].

**Scheme 5:**
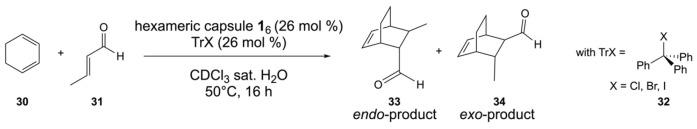
Diels–Alder reaction catalyzed by trityl halide in the resorcinarenyl capsule.

The stabilization of a chloride anion by the hexameric capsule was put to good use in the Diels–Alder reaction catalyzed by a trityl cation [100]. To this end, the group of Neri has carried out the reaction between 1,3-cyclohexadiene **30** and crotonaldehyde **31** in the presence of trityl halide **32** (26 mol%) and the resorcinarenyl capsule (26 mol%) in CDCl_3_ saturated with water. After 16 h at 50 °C, the conversions strongly depend on the nature of the halide. Conversions increased in the following order: I (no conversion) << Br (68%) < Cl (92%). In each case, the *endo*-product **33** was favored (*endo*-/*exo*-product ratio of 99/1) (Scheme 5).

**Scheme 6:**
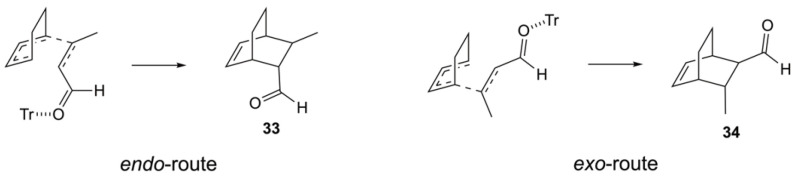
Endo- and exo-routes catalyzed with the trityl carbocation inside the capsule. The resorcinarene was not represented for clarity.

Molecular dynamics simulations and quantum mechanical calculations revealed that in the case of trityl chloride **32**, the encapsulation of the covalent form occurs through a hydrogen bond between one of the structural water molecules and the chlorine atom. This interaction makes the C-Cl bond weaker, thereby activating it and facilitating the formation of the ionic species. Once ionized, the chloride counterion displaces one of the water molecules bridging the capsule. A similar substitution of a water molecule was observed when the chloride ion was positioned outside the capsule before the simulation. In terms of trityl encapsulation, the three phenyl rings of the trityl group nestle into three distinct resorcin[4]arene cavities, establishing π•••π and CH•••π interactions. These in silico studies also explored the effects of different halides and counterions. When chloride was replaced by BF_4_^−^, the encapsulation of trityl remained similar to the chloride case. However, molecular dynamics simulations indicated that encapsulation occurred for bromide and iodide counterions, but the resulting ionic complex was unstable. After approximately 2 ns of simulation, the hydrogen bonding network broke down, leading to the disassembly of the capsule. This result is consistent with experimental observations of the absence or moderate conversions in the presence of iodide and bromide, respectively. To understand the regioselectivity of the reaction in favor of the *endo*-product **33**, the authors investigated the transition states leading to the *endo*- or *exo*-products using QM/SE methods performed using the ONIOM method incorporated in the Gaussian 16 package [94]. The transition states were found to be non-concerted within the capsule. Furthermore, the geometries of the transition state leading to the *endo*- and *exo*-products differed significantly between the free trityl BF_4_^−^ (without capsule) and the encapsulated trityl chloride. The energy difference between the transition states leading to the *endo*- (ΔG = 17.6 kcal/mol) and *exo*-products (ΔG = 20.2 kcal/mol) was notable, at 2.6 kcal/mol which correlates well with experimental findings, where the *endo*-/*exo*-products ratio exceeds 99/1 (Scheme 6).

**Scheme 7:**
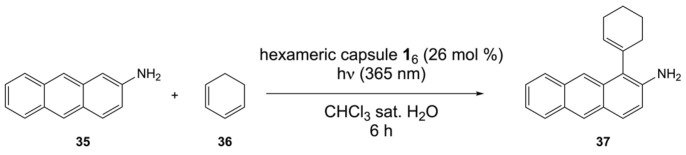
Alkylation of anthracen-2-amine **35** observed in presence of capsule and under irradiation.

In a recent article, the group of Neri explored the photoacidity of the hexameric capsule in the alkylation of anthracen-2-amine **35** with 1,3-cyclohexadiene **36** [101]. Under 365 nm irradiation and in the absence of the capsule, classical cycloadducts were observed. However, when the capsule was also present, a unique and new product was isolated in an 80% yield as a result of the alkylation of anthracen-2-amine **35** with 1,3-cyclohexadiene **36** (Scheme 7).

**Scheme 8:**
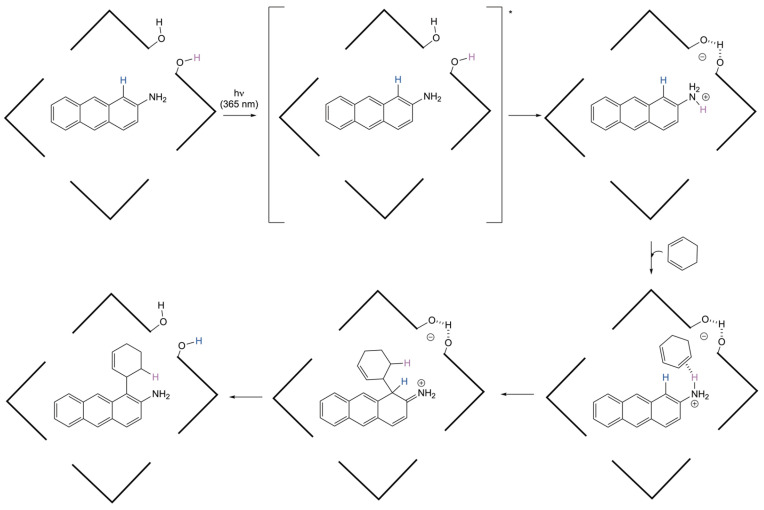
Proposed mechanism for the alkylation of anthracen-2-amine observed in the presence of a capsule under irradiation.

The formation of 1-(cyclohex-1-en-1-yl)anthracen-2-amine **37** was explained through DFT at the PBE0-D3/def2-SVP [75,102] with the NWChem package [77] and time-dependent DFT was performed with the ADF package [103] using the simplified TDDFT approach developed by Grimme [104]. First, the study of photochemical processes was conducted. The computational studies determined that photoexcitation of the capsule occurred at 301 nm, which is in excellent agreement with experimental data. Intersystem crossing to the T_1_ state is favored in this process, with an energetic gain of 14.9 kcal/mol. Proton transfer from the T_1_ state is more reactive than transfer from the S_0_ state, with a 19.7 kcal/mol energy penalty. DFT also verified the reduction of the pKa of the structural water molecules, leading to a lower value of approximately 4–5 pKa units. This value is consistent with the 8 log units reduction of the experimental pKa, estimated between −3.3 and −2.8. Subsequently, protonation of the amine by the capsule occurs, followed by deprotonation, which involves a reorganization of the hydrogen-bonding network. The induced charge is localized between two resorcinarenyl units, and the highest occupied molecular orbital of the capsule is located in this region. Two of the structural water molecules, which originally point inward within the capsule, reorient and form hydrogen bonds to stabilize the capsule. The activated reactant then proceeds to react, as shown in Scheme 8.

**Scheme 9:**
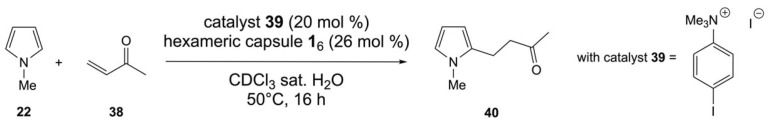
Michael reaction between *N*-methylpyrrole **22** and methyl vinyl ketone **38**.

In this last example, Neri and co-workers investigated the Michael reaction between *N*-methylpyrrole **22** and methyl vinyl ketone **38**. The authors used an “amplified halogen bonding catalyst”, namely the 4-iodo-*N,N,N*-trimethylbenzenaminium iodide **39** (Scheme 9) [45]. This organocatalyst is unique because it has a trimethylammonium group that can establish π•••cation interactions with the inner wall of the cavity. It also has an iodophenyl moiety that donates to noncovalent interactions. In the optimal catalytic conditions, the product of Michael reaction **40** was isolated in 98% yield. This was performed in CDCl_3_ saturated with water at 50 °C for 16 h. Once again, without the presence of the supramolecular capsule, no conversion of *N*-methylpyrrole **22** was observed.

**Figure 7:**
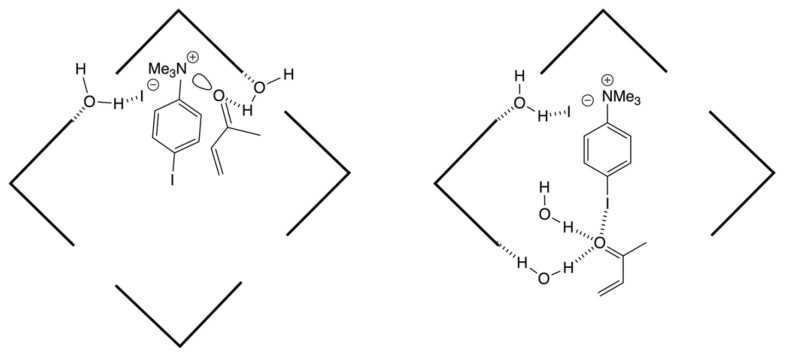
Transition states involving the hexameric capsule (**left**) and the pentameric assembly (**right**).

In silico studies were conducted to understand the role of the halogen-bonding interaction in the activation of methyl vinyl ketone **38**. Quantum chemical investigations were performed using the ONIOM method (M06-2X:PM6) [60,105]. The calculations show that the sequence of reactant encapsulation occurs in the following order: the organocatalyst **40,** for which the counteranion iodide is located inside the capsule through hydrogen-bond interactions with a bridging water molecule; the methyl vinyl ketone **38**; and the *N*-methylpyrrole **22**. In the case of a hexameric capsule, no transition state involving halogen bonding that could lead to the product was found. However, they discovered that in the presence of a pentameric capsule **1**_5_, a transition state involving halogen bonding between the reactants, capable of leading to the desired reaction, was indeed present (Figure 7).

**Scheme 10:**
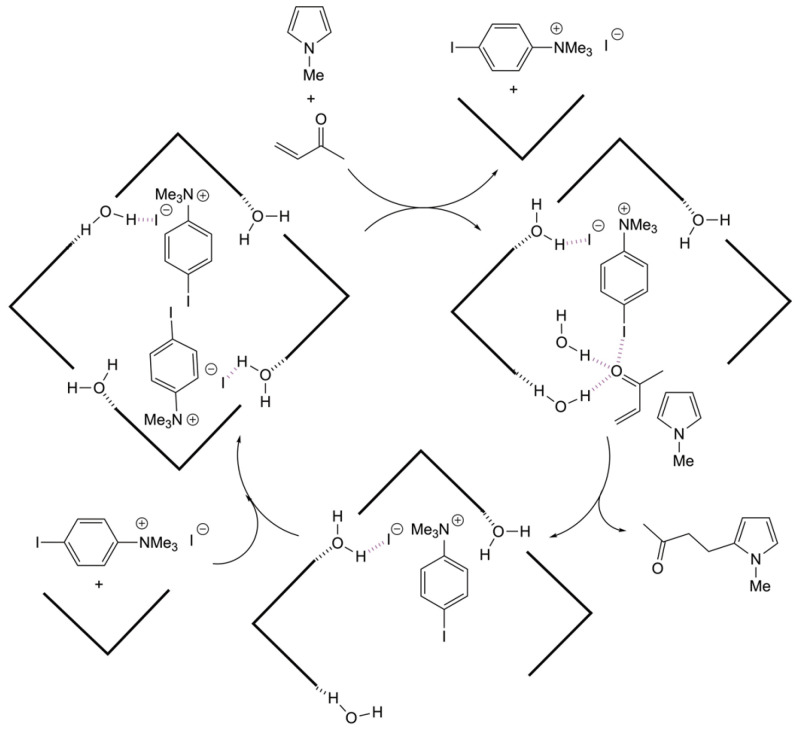
Postulated mechanism involving the pentameric assembly **1**_5_.

The theoretical study of the organic reaction was conducted in an open pentameric capsule **1**_5_, a significant departure from previous methods. In this open environment, two hydrogen bonds were observed between the carbonyl oxygen of methyl vinyl ketone **38** and a structural water molecule, as well as one encapsulated within the pentameric assembly. This synergistic interaction of halogen and hydrogen bonding activates the carbonyl group, facilitating the reaction. The pentameric capsule also stabilizes the counterion through hydrogen bonds with structural water molecules. Another transition state involving the hexameric capsule was identified, but it was found to be 6.28 kcal/mol higher in energy than the previous pentameric-based transition state. Experimental validation of the formation of the pentameric capsule during the reaction was achieved through DOSY NMR and Fourier transform ion cyclotron resonance (FT-ICR) mass spectrometry. At the end of the reaction, the thermodynamically more stable hexameric species is reformed (Scheme 10). Further studies on alternative counterions were also conducted, with experimental observations in agreement with their theoretical results. Additionally, a second-order perturbation theory analysis (SPOT) [106] indicates that the reaction is polar in nature. Reactivity is thus correlated with the local electrophilicity of the terminal carbon of the vinyl group in methyl vinyl ketone. Higher values of local electrophilicity were observed in the transition states associated with the formation of the pentameric capsule, demonstrating the enhanced catalytic activity of this particular capsule form.

### 2.2. Pyrogallol[4]arene

**Figure 8:**
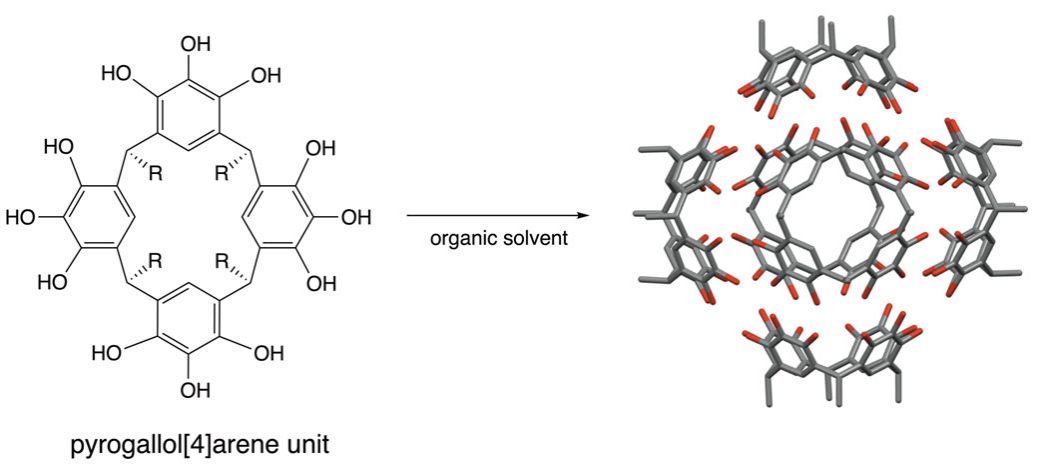
Pyrogallol[4]arene assembly formed from 6 units of pyrogallol[4]arene in organic solvent.

In 1999, Mattay and co-workers showed that 2,8,14,20-tetra-*iso*-butyl-5,11,17,23-tetrahydroxyresorcin[4]arene **2** leads to the formation of a hexameric assembly in both the solid state using X-ray diffraction and in solution (Figure 8) [107]. Subsequent studies showed that hexameric assembly of pyrogallol[4]arene remains stable in solution [23,108,109]. The self-assembly of this capsule does not require the presence of water, as it is stabilized by an intricate network of 72 hydrogen bonds formed between the phenolic units of the macrocycles [110]. This arrangement creates a cavity of approximately 1400 Å^3^, enabling the study and characterization of several encapsulation examples. However, its catalytic efficiency is diminished compared to its resorcinarene counterpart, as will be detailed later. This section is divided into three subsections. The first subsection is a study on the early-stage formation of the capsule. The second subsection is devoted to the examination of various encapsulation studies. The third subsection is the sole documented example of catalysis occurring within this self-assembled system.

#### 2.2.1. Capsule Assembly

Capelli and Piccini conducted a detailed in silico study on the formation of pyrogallolarene assemblies [47]. The authors performed metadynamic simulations using the same methodology as for the resorcinarene-based hexameric capsule previously described *vide supra*. The only difference was that the calculations with pyrogallol[4]arene were performed in anhydrous chloroform. While the formation of the hexamer is thermodynamically favorable, the free energy surfaces associated with the formation of the pyrogallolarene assembly differ significantly from those of the resorcinarene counterpart. Firstly, the formation of a pyrogallolarene dimer **2**_2_ is not thermodynamically favorable, unlike the resorcinarene dimer **1**_2_. Several factors contribute to this difference. The additional hydroxyl group in pyrogallolarene partially hinders the formation of intermolecular hydrogen bonds in the dimer species. Pyrogallolarene is known to form eight intramolecular hydrogen bonds, which limits its capacity for intermolecular interactions. The team calculated the probabilistic distribution of intermolecular hydrogen bonds within the pyrogallolarene dimers and observed a sharp distribution corresponding to a single intermolecular hydrogen bond. In contrast, resorcinarenes, with an average of four intramolecular hydrogen bonds, exhibit a broader distribution of intermolecular hydrogen bonds, ranging from one to five. Additionally, the formation of pyrogallolarene dimers introduces unfavorable dipole–dipole interactions, as demonstrated in their study. The first metastable structure observed on their free energy surfaces is a trimer **2**_3_ described as a “cup-shaped half of a hexamer”. Subsequently, the formation of a pentameric **2**_5_ species is observed, though it is relatively less stable than the trimer, followed by the assembly of the hexamer **2**_6_. In this system, the equilibrium predominantly lies between the monomer **2** and the hexamer **2**_6_.

The authors also studied the encapsulation dynamics of solvent molecules within the pyrogallolarene hexamer. Their findings suggest an average dynamic encapsulation of 9–10 molecules, which is significantly higher than the 7–8 molecules predicted by NMR studies [111,112]. This example also shows that the number of solvent molecules encapsulated by NMR appears to be minimized. This discrepancy, compared to the resorcinarene assembly, is here attributed to the lesser steric constraints imposed by the absence of water molecules, a conclusion supported by their analysis.

#### 2.2.2. Formation of Inclusion Complexes

**Figure 9:**
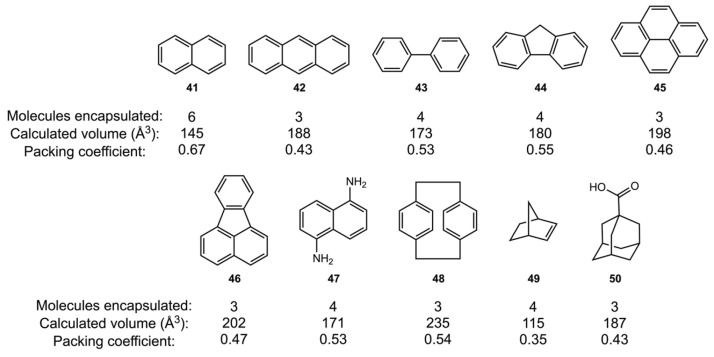
Molecules studied in silico with the maximal number encapsulated inside the pyrogallolarene hexamer, the calculated volume (Å^3^) and the corresponding packing coefficient.

Purse and co-workers extensively investigated the encapsulation of a wide variety of guest molecules, including arenes, alkanes, amines, and carboxylic acids. The authors employed the melting approach, a solvent-free method [113,114] and they used molecular modeling with HyperChem 8.0.6 and Spartan 04 and by minimizing the guests on the interior at the AM1 level of theory. This allowed them to determine the host–guest stoichiometry and calculate the packing coefficient for each system [115,116]. Their capsule models were based on an X-ray structure, which served as a framework for the simulations. These simulations provided insight into the arrangement of guest molecules within the capsule. The details regarding the studied molecules **41**–**50**, their stoichiometry, and packing coefficients are illustrated in Figure 9. Computational studies indicate that the encapsulation of these guest molecules adheres to the optimal packing coefficient defined by Rebek, which lies within the range of 0.55 ± 0.09 [86]. Naphthalene **41** (0.67) had the higher value, which can be rationalized by its small size and its efficient packing within the capsule cavity. Multiple π•••π interactions provide further stabilization. In contrast, the lowest packing coefficient was found for norbornene **49** (0.35). This guest lacks stabilizing π•••π interactions and exhibits a molecular geometry that is poorly suited for structural adaptation within the capsule (lock-and-key principle) [117], as seen in the simulation, thereby precluding a more favorable packing efficiency.

**Figure 10:**
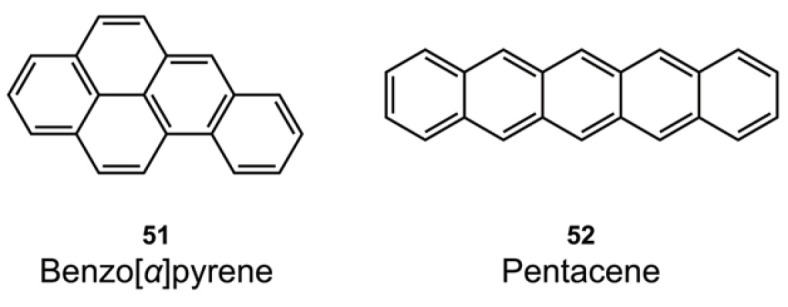
Studied molecules in silico by the group of Atwood inside the pyrogallolarene assembly.

Atwood and co-workers explored two examples of polycyclic aromatic hydrocarbon encapsulations, **51** and **52**, as shown in Figure 10 [118]. While X-ray structures of the corresponding supramolecular assemblies were obtained, they did not provide direct visualization of the guest molecules within the confined space. Several factors contributed to this limitation. First, significant disorder within the cavity obscured the positioning of the encapsulated species. Second, the guest molecules could adopt various orientations relative to the macrocyclic subunits. Finally, the occupancy rate of the capsules was low. For instance, benzo[*α*]pyrene **51** was encapsulated in only 1 out of 14 capsules, while pentacene **52** showed an even lower rate of 1 in 40. To overcome these challenges, the authors employed molecular modeling with B3LYP/cc-pVDZ calculation [119] to study the latter inclusion complexes. The models revealed that pentacene **52** could only fit within the cavity when positioned between two macrocyclic subunits. In contrast, benzo[*α*]pyrene **51** exhibited a greater range of possible orientations within the capsule. However, the large size of the molecule prevented it from adopting the optimal arrangement for maximizing interactions, such as aligning along the capsule walls, as seen in previous studies for a similar molecule [120]. Instead, the study observed that benzo[*α*]pyrene **51** was tilted and projected partially outside the half-nanocapsule, forming possible CH•••π and CH•••O interactions.

The analogous resorcinarene **1** is well known for its ability to encapsulate ammonium ions, but the pyrogallolarene **2** has apparently struggled to achieve the same. It was only observed after 17 years in benzene [121]. This discrepancy was initially puzzling, given the electron-rich cavity provided by the pyrogallolarene capsule. Tiefenbacher and co-workers set out to address this question, and their findings on the resorcinarene system are discussed in the previous section. Their DFT studies demonstrated that the pyrogallolarene capsule can also stabilize ammonium ions [79]. Their results indicate that the pyrogallolarene capsule can stabilize ammonium ions as effectively, if not more so, than its resorcinarene counterpart. Specifically, calculations on NEt_4_^+^ **53** showed greater stabilization with the pyrogallolarene capsule, with energy differences ranging from approximately 4 to 12 kcal/mol. In contrast, the stabilization of anions differed significantly between the two systems, which provided a key explanation for the observed behavior. The pyrogallolarene capsule is simply incapable of effectively stabilizing anions. The group of Tiefenbacher calculated the electrostatic potential map of the inner surface of the pyrogallolarenyl capsule to illustrate this point. The results revealed a critical difference: unlike the resorcinarene **1**, the pyrogallolarene **2** lacks regions of high positive potential on its inner surface, preventing efficient anion stabilization. Further DFT studies on the encapsulation of NEt_4_Br showed that, even if the pyrogallolarene capsule stabilizes the cation **53** more effectively, the overall stabilization of the species is weaker (3–9 kcal/mol) due to the non-stabilizing effect of the anion. As demonstrated by NMR spectroscopy in the case of the [EtNPr_3_][MeSO_3_] salt **54**, the counteranion remained external to the cavity, incurring a charge separation cost (in CDCl_3_). These findings highlight the delicate balance between cation and anion stabilization within these systems and underscore the electrostatic characteristics of the pyrogallolarenyl capsule.

#### 2.2.3. Study of Mechanisms Inside the Pyrogallolarenyl Capsule

The pyrogallolarenyl capsule **2**_6_ has far fewer applications in catalysis than its resorcinarene **1**_6_ counterpart. This limitation stems from its apparent lack of catalytic properties. As described in the previous section by Tiefenbacher and co-workers, the pyrogallolarenyl capsule poorly stabilizes counteranions, resulting in weaker stabilization of confined species and their intermediates [79]. Additionally, the pyrogallolarenyl capsule has a lower acidity, measured at a pKa of 9.5–10, which is four pKa units higher than the resorcinarenyl capsule. This lower acidity further limits its catalytic potential. DFT calculations confirm this acidity, showing that the proton affinity of the resorcinarene species is approximately 5 kcal/mol lower than that of pyrogallolarene. Many catalytic applications of the resorcinarene capsule rely on its acidity and bridging water molecules. In the pyrogallolarene assembly, this effect is less pronounced due to its lower acidity and the absence of water molecules.

**Scheme 11:**
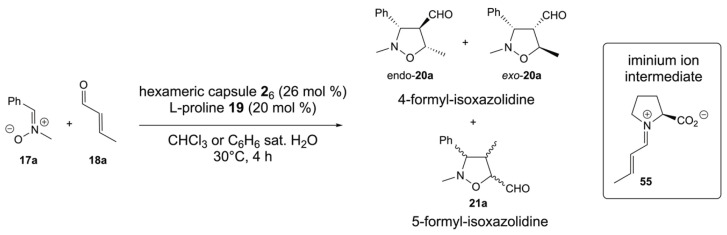
Model reaction of the 1,3-dipolar cycloaddition and the iminium intermediate studied in silico.

However, a recent publication by the group of Neri reported the unique use of the pyrogallolarene capsule in catalysis [122]. In their study, they investigated the 1,3-dipolar cycloaddition between nitrone **17a** and crotonaldehyde **18a** in the presence of L-proline **19** (Scheme 11). Preliminary observations revealed that chloroform molecules compete with the encapsulation process within the pyrogallolarenyl capsule. This suggests that encapsulation of the guest and catalysis within the supramolecular capsule is unfavorable. However, using benzene, a less competitive solvent, improved encapsulation of the iminium **55** and nitrone **17a**, enabling catalytic activity. When the reaction was carried out in CHCl_3_, a competitive solvent to the encapsulation of the substrates, the isoxazolidine **20a** products were isolated with a yield of 62%, and the *exo*-product was favored (60% of the formed products) among the eight possible products *vide supra*. In contrast, when the reaction was carried out in the confined space of the resorcinarenyl capsule with a non-competitive solvent such as benzene, the *endo*-product of the 4-formyl-isoxazolidine **20a** predominated (58% of the formed products). The authors demonstrated by NMR spectroscopy that when the reaction is carried out outside the capsule, the formation of the *exo*-product **20a** is favored (use of CHCl_3_ as solvent). Conversely, when the reaction is carried out in the inner space of the capsule, the reaction leads mainly to the *endo*-product **20a** (use of benzene as solvent).

To support these observations, quantum-mechanical investigations were performed in the pyrogallolarenyl assembly. When the simulations were realized with benzene as solvent, two assemblies between the capsule and iminium ion **55** were stable, inside and outside the pyrogallolarenyl capsule. In both cases, the iminium ion was immobilized by two hydrogen bonds formed between the carboxylate group of the iminium ion **55** and a pyrogallolarene subunit. Furthermore, calculations show that the inclusion complex with the iminium ion **55** inside the capsule is more stable by 10.7 kcal/mol. However, this stabilization was only observed when the capsule was occupied by non-competitive species, which is not the case when using chloroform or competitive guests. The complexation of nitrone **17a** and iminium **55** is exergonic, and the process is significantly more exergonic inside the capsule than outside. The authors then investigated the mechanism pathway. Eight reactive pathways were identified. In both the *meta* and *ortho* cases, stable initial complexes were observed at the start of the reaction. The *meta* pathway was unequivocally the most favorable reaction channel within the capsule. Furthermore, the two transition states were almost concerted, resembling a Michael-type addition. These results provide a comprehensive understanding of the mechanism of the reaction, highlighting the role of encapsulation in selectively stabilizing reactive intermediates and favoring specific reaction pathways. The modeling in chloroform revealed that the reactive iminium **55** intermediate resides outside the capsule. This stable intermediate forms a hydrogen bond between its carboxylate group and one of the hydroxyl groups of the capsule, similar to observations in the benzene simulations. After calculating the activation energies for reactions occurring both inside and outside the capsule, the authors estimated the relative contributions of each pathway. These calculations are in close alignment with the experimental results for the reaction in chloroform. This suggests that when a guest, such as chloroform, partially competes with the reactants, the reaction proceeds simultaneously inside and outside the capsule, albeit with varying proportions of encapsulated and free reactive intermediates. Interestingly, compared to the same reaction carried out using the resorcinarenyl capsule, the pyrogallolarenyl capsule exerts a significantly greater influence on stabilizing the reactive intermediates. These findings underscore the pivotal role of solvent choice in modulating the reactivity and selectivity of catalytic systems involving the pyrogallolarenyl capsule.

### 2.3. Velcrand

**Figure 11:**
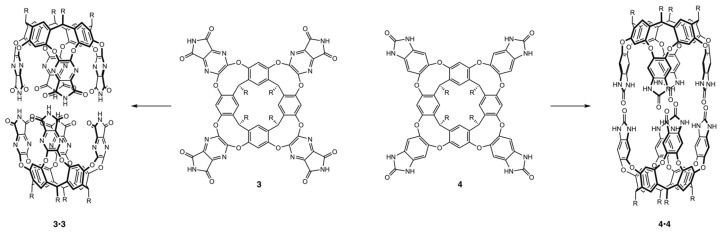
Imide-velcrand **3** and urea-velcrand **4** described in the studies. Feet are not represented and depend on the desired solubility. In the simulation, they are simplified as methyl feet to avoid computational time, as they do not affect the cavity and encapsulation properties.

When adjacent phenols of the aromatic ring of the resorcin[4]arene are bridged by aromatic moieties, the formed cavitands exist in two forms: the receptive vase shape and the unreceptive kite (or velcrand) [123]. These compounds exist in dimeric form and were called «velcraplexes» by Cram [124]. Several velcrands have been studied to date, with two representative types illustrated in Figure 11. Velcrand **3** was the first to be synthesized by Rebek and co-workers in 1998 [125]. Eight bifurcated hydrogen bonds hold this capsule up. The cavity of this structure, as calculated through energy-minimized simulations (MacroModel 5.5, Amber* force field) [126], measures 425 Å^3^, enabling the investigation of guest encapsulation.

**Figure 12:**
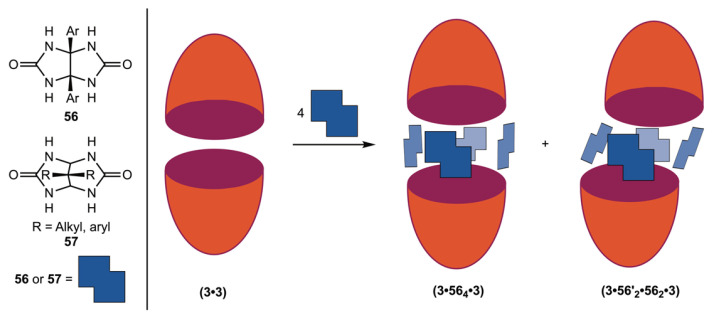
Spacers studied in this review (**left**) and view of the extended capsule **3**•**56**_4_•**3** (**right**).

The present review focuses on imide-velcrand **3** and urea-velcrand **4**, which exhibited comparable cavity volume [127]. The dimensionality of these assemblies can be further extended by incorporating spacers, as shown in Figure 12 [128,129]. For instance, the inclusion of four spacers **56** results in the formation of two isomers of the capsule **3**•**3** with a stoichiometry of **3**•**56**_4_•**3**, as depicted in Figure 12.

These novel assemblies possess larger cavities, approximately 620 Å^3^, as determined through in silico calculations. In the first extended isomer form (denoted as **3**•**56**_4_•**3**), the glycoluril units form hydrogen bonds with both macrocycles and their neighboring glycoluril units. In the alternative form (denoted as **3**•**56**_2_•**56′**_2_•**3**; the prime “ ‘ “ denotes the different orientation of the spacer), two glycoluril units establish hydrogen bonds with both macrocycles and their neighbors, whereas the other two glycoluril units bond only with one cavitand and its respective neighbors. This second isomer is calculated to be more stable by 2 kcal/mol, according to M06-2X/6-31G(d,p) level of theory [73,105,130]. Studies have also focused on the host–guest properties of these assemblies, with some particularly using theoretical approaches, which are discussed in this section.

#### 2.3.1. Encapsulation of Guests

Rebek and co-workers studied the encapsulation of a series of alkanes in the gas phase within the imide-velcrand (**3**•**3**) and its glycoluril extended capsule (**3**•**56**_4_•**3**) [131]. The alkanes studied included ethane **58**, cyclopropane **59**, *n*-butane **60**, and *n*-hexane **61**. The authors calculated the van der Waals volumes of these molecules in silico using GRASP [132] and determined the packing coefficients based on the number of guests within the two different capsules at 295 K and room temperature (Table 1). The results are consistent with those of Kitaigorodsky, with packing coefficients below 51% for gaseous-phase guests [133], and stable inclusion complexes were observed when approximately 40% of the inner space of the capsule was occupied.

A similar work was carried out with straight-chain alkanes C_10_ to C_15_ within velcrand (**3**•**3**) [134]. The modeling procedures were conducted in a manner consistent with the previous example. This enabled the calculation of molecular volumes based on their conformations (either extended or folded) and their corresponding packing coefficients. Simulations further determined the internal length of the capsule to be approximately 16 Å. In an interesting way, *n*-tetradecane **62** (C_14_H_30_), which measures 20 Å in its linear form, was successfully encapsulated. This indicates that the molecule adopts a helical conformation, a shape that maximizes CH•••π interactions with the inner aromatic rings of the capsule. This hypothesis was confirmed through NMR spectroscopy and computational simulations. This is in contrast to alkanes in solution, where the linear conformation is predominant. Once inside the capsule, at least five gauche interactions, each of 0.5 kcal/mol, exist at both ends of the *n*-tetradecane **62** chain. On the NMR time scale, a rapid racemization of the enantiomeric helical forms occurred with a barrier of about 2.7 kcal/mol for each gauche bond [134,135].

**Figure 13:**
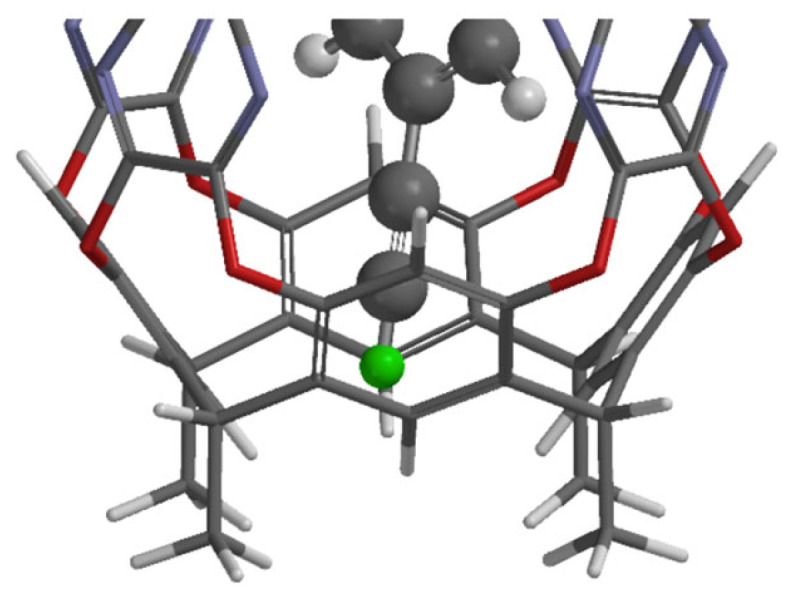
View of the acetylenic function (hydrogen in green) in proximity to the inferior ring of the resorcinarene moiety.

A difference in encapsulation behavior emerged between *n*-pentadecane **63** and 1-pentadecyne **64**. In the case of the acetylenic derivative, a particular positioning of the terminal alkynes was observed near the inferior rim of the macrocycle. This arrangement, for steric reasons, could not be achieved with methyl substituents. NMR investigations and DFT-based calculations of natural chemical shift index values for the magnetically shielded regions of the capsule at the B3LYP/6–31G* level of density functional theory [72] were in good agreement. This confirmed the spatial interactions of the terminal acetylenic moiety within the velcrand (Figure 13). NMR studies and modeling revealed that bulkier guests caused the alkyne group to approach closer to the inferior rim. Additionally, semi-empirical energy minimization at the AM1 level of theory [116] structure revealed the positioning of the guest. The acetylenic function approached the inferior rim, and the *n*-butyl chain adopted a gauche conformation, minimizing the steric constraints imposed by encapsulation [136].

In the case of water-soluble urea-velcrand (**4**•**4**), in which water-soluble feet are grafted to the macrocycle instead of undecyl chains, investigations have shown that the capsule can adapt to guest molecules [137]. For instance, with *n*-heptadecane (C_17_H_36_) **64** encapsulation, computational studies at the ab initio level (HF/6-31G*) basis set with Gaussian 09 software [59] showed the incorporation of four water molecules into the hydrogen-bonding network within the capsule. This inclusion extended the internal volume of the capsule, which, under water-free conditions, is comparable to that of velcrand (**3**•**3**) and would not initially seem capable of accommodating a molecule of this size. The water molecules also facilitated better accommodation of the guest within the cavity, aligning the aromatic walls to conform to the folded alkane. However, they also noted that while adding more water molecules could provide additional space for guest motion, it would simultaneously weaken the favorable CH•••π interactions between the host and the guest.

As mentioned earlier, the self-assembly capsule based on imide-velcrand (**3**•**3**) can incorporate spacers between the two macrocycles, effectively increasing its volume. A study of the group of Rebek investigated imide-velcrand (3•3) in the presence of **57** as spacers [138]. The authors observed that, according to the guest, new supramolecular assemblies containing a greater or lesser number of spacers **57** could be formed. These assemblies were characterized using NMR spectroscopy, confirmed by DFT-level NMR spectral simulations (B3LYP/6-31G*), and analyzed via molecular modeling using Spartan [73]. The simulations validated the observed symmetries, chirality of the capsules, and the NMR chemical shifts.

**Figure 14:**
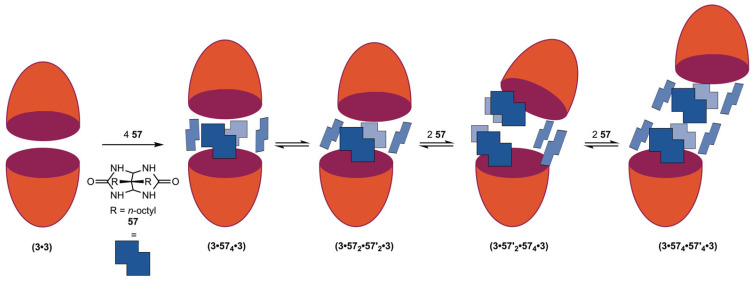
Formation of the extended capsule from **3**•**3** and **57**.

Two types of assemblies were observed in the presence of *n*-tetradecane (C_14_H_30_) **62**, **3**•**57**_4_•**3** and **3**•**57**_2_•**57′**_2_•**3** (the prime “ ’ “ denotes the different orientation of the propanediurea carbonyls of the spacer **57**). The former is a linear “belt” shape with a *D*_4_-symmetric structure, and the latter is a twisted “belt” shape with a *C*_2h_-symmetric structure (Figure 14). These two assemblies are in equilibrium, with the balance shifting toward the formation of **3**•**57**_4_•**3** when *n*-tetradecane (C_14_H_30_) **62** is encapsulated and toward the formation of **3**•**57**_2_•**57′**_2_•**3** when *n*-heptadecane (C_17_H_36_) **64** is hosted. The transition from **3**•**57**_4_•**3** to **3**•**57**_2_•**57′**_2_•**3** is attributed to an accessible cavity length that is approximately 1 Å longer in the case of the less symmetrical assembly (**3**•**57**_2_•**57′**_2_•**3**). This length fits better with bulkier guests. The study of encapsulation of alkanes with an increasingly long carbon chain, from *n*-octadecane (C_18_H_38_) **65** to *n*-tricosane (C_23_H_48_) **66**, has revealed the formation of new supramolecular structures with “S” and “banana” shapes corresponding to the **3**•**57′**_2_•**57**_4_•**3** and **3**•**57**_4_•**57′**_4_•**3** structures, respectively (Figure 14). For these different capsules, the packing coefficients of the guests, from *n*-C_14_H_30_ **62** to *n*-C_23_H_48_ **66**, were calculated. The results indicate that the formation of these capsules is consistent with packing coefficients close to the ideal value of 55% [86] (Table 2). The formation of inclusion complexes was confirmed by evaluating the positioning of the guest in the cavity through simulations. The assemblies were energy-minimized with the guest inside at the molecular mechanics level of theory (Spartan 03), and the cavity volumes were subsequently determined using Swiss-PdbViewer 4.0.1.

#### 2.3.2. Co-Encapsulation of Guests

An illustrative example from Wang and co-workers focused on the co-encapsulation of dichloromethane and various organic molecules within imide-velcrand (**3**•**3**) [139]. This study examined the co-encapsulation of cyclohexane **67**, methylcyclohexane **68**, 1,3-dioxane **69**, and hydroxycyclohexane **70** using DFT. The structures were optimized utilizing Perdew–Wang’s exchange and correlation functionals (PW91) in conjunction with the 3-21G basis set [140]. All computations were performed using the Gaussian 03 software package [59]. The results are clear: when dichloromethane is co-encapsulated with an organic molecule, the host–guest complexes formed are more stable than systems in which only the organic molecule is encapsulated. This increased stability is due to the internal volume of the capsule being more efficient, allowing the guest molecules to occupy regions that stabilize them. Notably, the study revealed the presence of hydrogen bonding between the oxygen atom of 1,3-dioxane **69** and an imide group of the capsule (**3**•**3**) in the case of 1,3-dioxane associated with dichloromethane. These findings underscore the efficacy of co-encapsulation in optimizing spatial and interaction dynamics within the velcrand system, paving the way for more efficient host–guest assemblies.

**Figure 15:**
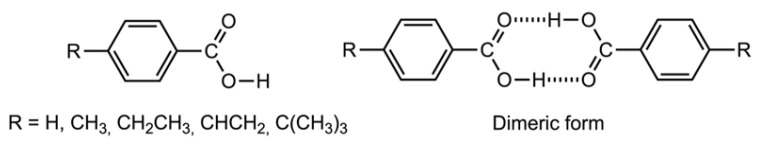
Studied guests inside **3**•**3**, **3**•**56**_4_•**3** and **4**•**4** capsules.

The group of Theodorakopoulos has investigated in silico several examples of inclusion complexes in which two carboxylic acids, in dimeric form, are hosted in the **3**•**3**, **3**•**56**_4_•**3** or **4**•**4** capsules [141]. Their results are directly comparable with experimental observations, as the dimers are sufficiently stable to be directly observed by NMR spectroscopy [142]. Initially, the study focused on benzoic acid **71**, *p*-methyl-benzoic acid **72**, *p*-ethyl-benzoic acid **73**, *p*-vinyl-benzoic acid **74** and *p*-*tert*-butyl-benzoic acid **75**, which are known to form dimers. The encapsulation complexes were optimized by DFT calculations employing the M06-2X [130] functional in conjunction with the 6-31G(d,p) basis set [73]. For all structures determined, basis set superposition error corrections to the dimerization energy have been taken into account using the counterpoise procedure [143]. All calculations were performed using the Gaussian 09 program package [59]. This method allowed for a comprehensive examination of various homodimers of carboxylic acids, both in their free state (in solution) and encapsulated form (see Figure 15 for a visual representation of the guests). The primary goal was to elucidate the effects of encapsulation-induced compression on these dimers.

In silico investigations revealed that the dimerization energy decreases slightly upon encapsulation compared to the free-solution case (in the range of −14 to −17 kcal/mol), except for the benzoic acid substituted with an alkyl chain (CH_3_ and CH_2_CH_3_) (**72** and **73**) in the **3**•**3** capsule. In these cases, the significant compression within the cavity disrupts the dimer of acids. The decrease in dimerization energy, the smallest elongation of the OH•••O hydrogen bond, is attributed to stabilizing interactions between the encapsulated species and the capsule, mainly through hydrogen bonds between the two carboxyl groups of the acids and the terminal hydrogen and oxygen atoms of the two imide-velcrands **3**•**3**. These interactions make encapsulation energetically favorable in all cases, even in the absence of a dimer with alkyl substituents. In these latter cases, due to confinement effects, the guests exhibit a stacking arrangement, with one positioned above the other. In this position, the OH•••O hydrogen bond distances of the encapsulated “dimer” increased from 1.66 Å in the free dimer to 2.56–3.72 Å in the capsule. As experimentally observed [144], the compression of the hosted dimer by the walls of the capsule **3**•**56**_4_•**3** reduced the dimerization energies, accompanied by shorter hydrogen-bond lengths, with the exception of *p*-*tert*-butyl-benzoic acid **75**. Despite the energy required to induce this compression, the encapsulation of the dimeric guest was energetically favorable. Additionally, while the free dimers are coplanar in solution, the encapsulated dimers exhibit deviations from coplanarity. This effect diminishes in the larger capsule, **3**•**56**_4_•**3**, where spatial constraints are reduced. Finally, the study found good theoretical and experimental correlations between the bond lengths in the O-H•••O network, highlighting the agreement between computational predictions and experimental measurements.

Theodorakopoulos, Rebek and co-workers further explored the distribution of encapsulated homodimers and heterodimers of carboxylic acids and amides within velcrands **3**•**3** and **3**•**56**_4_•**3** [145,146]. For the inclusion complexes made with benzamide **76**, either as a homodimer (benzamide–benzamide) or heterodimer (benzamide–benzoic acid) and the smaller capsule **3**•**3**, the formation of zero and a hydrogen bond, respectively, between the dimers and the capsule was observed. This is in contrast to the larger capsule, **3**•**56**_4_•**3,** in which two supramolecular interactions were present. The authors integrated energetic data with statistical factors to calculate the percent distribution of the different dimers, homo and hetero, encapsulated in both capsules. For instance, in the smaller capsule **3**•**3**, the calculated distribution of the inclusion complexes hosted benzamide–benzamide, benzamide–benzoic acid and benzoic acid–benzoic acid dimers, which were found to be 18, 35 and 47%, respectively. These values are in agreement with experimental distributions of 19, 22 and 59%, respectively [147].

**Figure 16:**
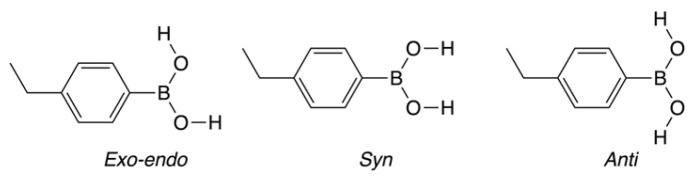
Three possible conformations of the boronic acid **79**.

The authors also investigated the distribution of homodimers and heterodimers in a mixed system of *p*-ethyl-benzoic acid (**77**), *p*-ethyl-benzamide (**78**), and (*p*-ethyl-phenyl)boronic acid (**79**). They studied both free molecules in solution and those encapsulated [142]. Theoretical work was carried out using DFT calculations under various conditions: gas phase, solvated in CCl_4_, in DMF, and within capsules **3**•**3**, **3**•**56**_2_•**56′**_2_•**3** and **3**•**56**_4_•**3**, in which, for the last two capsules, the four glycoluril molecules were stitched by 24 and 32 hydrogen bonds. In the theoretical study, only the latter structure involving 32 hydrogen bonds was employed. Optimization of the encapsulated structures was problematic for the capsules, which had 24 hydrogen bonds. With these guests, two configurations, *cis* and *trans*, exist in the dimeric forms. The capsule has slightly lower interaction energy, up to 0.5 kcal/mol, for the *trans*-configurations of the dimers. For the (*p*-ethyl-phenyl)boronic acid **79**, three possible configurations were considered: *exo*-*endo*, *syn* and *anti* (Figure 16). The computational results indicate that the *exo*-*endo* configuration was the most stable, regardless of whether the dimers are homodimeric or heterodimeric.

Gas-phase calculations indicate the stability order for non-encapsulated dimers: **79**•**79** > **78**•**79** > **78**•**78** > **77**•**79** > **77**•**78** > **77**•**77** in the range 10.6 to 17.1 kcal/mol (calculated with M06-2X/6-31G(d,p) at various levels of theory). In CCl_4_, the only difference was that **78**•**78** and **77**•**79** became degenerate. When the calculation was carried out in DMF, the **78**•**78** dimer became the least stable. For encapsulated dimers, the authors used a combination of computational methods, including DFT at the M06-2X/6-31G(d,p) level and ONIOM approaches [M06-2X/6-31G(d,p); PM6] and [MP2/6-31G(d,p); PM6] using the Gaussian 09 program package [73,105,130,148,149,150,151]. Both methods produced nearly identical dimerization energies, though some convergence issues arose for specific encapsulated configurations. The dimerization energy order remained similar to the gas-phase calculations: **79**•**79** > **78**•**79** > **78**•**78** > **77**•**79** > **77**•**78** > **77**•**77**. The interaction energy between the dimers and the capsule contributed significantly to the overall stabilization of encapsulated dimers. When the total encapsulation energies were summed, the energetic hierarchy of encapsulated dimers shifted, resulting in a new order, denoted as **77**•**77** > **77**•**79** > **78**•**79** > **78**•**77** = **79**•**79** > **78**•**78** (Table 3).

The authors used theoretical data to determine the relative distributions of dimers in mixed systems (**78** + **79**, **77** + **79**, **77** + **78** and **77** + **78** + **79**) under both free and encapsulated conditions. The predicted distributions closely matched the experimental observations, as shown by incorporating statistical factors. For example, when the three guests were mixed, the calculated (ONIOM-MP2/6-31G(d,p)) distribution of the encapsulated dimer was **77**•**77**, **78**•**79**, **77**•**79**, **79**•**79**, **78**•**77** and **78**•**78, which were** 29, 20, 17, 11, 15 and 9, respectively, compared to 34, 23, 18, 15, 6 and 4, respectively, for the experimental values [152]. The enhanced prevalence of boronic acid dimer **77**•**77** is attributable to its adaptable structure.

It is interesting to note that for their calculations, the authors compared computational methods, which provided comparable results. Nevertheless, ONIOM calculations were approximately 30 times faster, making them advantageous for larger systems. For the systems studied here, employing PM6 at the ONIOM lower level proved sufficient when paired with higher-level calculations such as M06-2X/6-311+G(d,p) or MP2/6-311+G(d,p).

### 2.4. Octa Acid

**Figure 17:**
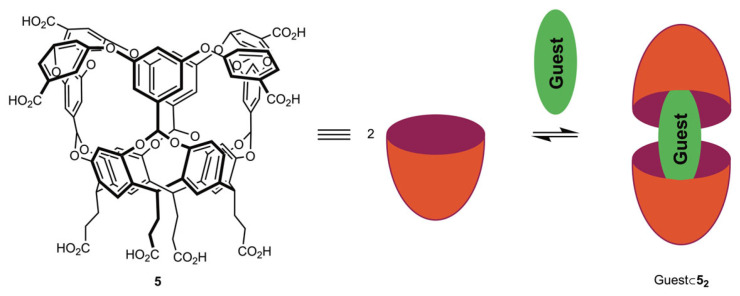
Octa acid capsule forms in the presence of a guest.

A notable example of a supramolecular assembly based on resorcinarene is the cavitand illustrated in Figure 17. This macrocyclic molecule has four carboxylic moieties attached to the larger and smaller rims of the macrocyclic platform. It is commonly referred to as “octa acid”. This macrocyclic system spontaneously forms dimers, also known as capsuleplexes, in the presence of hydrophobic guests, forming a supramolecular interaction network [153]. The primary driving force for encapsulation is the hydrophobic effect, and additional stabilization is provided by π•••π interactions, CH•••π interactions, and van der Waals forces [154,155,156]. A key advantage of this capsule is its solubility in aqueous solutions under basic conditions (pH ~9, achieved using a borate buffer). This solubility arises from its eight carboxylic acid groups of each cavitand, allowing it to form inclusion complexes with host/guest ratios of 2:1 or 2:2 [157]. Despite the aqueous medium, the interior of the capsule remains hydrophobic [153]. The assembly of macrocycles with host molecules is stable on the millisecond scale, although the host molecules undergo a fast rotation along the molecular x-axis [158,159].

The confined environment provided by the octa acid capsule introduces several key effects for the study of excited states: solvent isolation, reagent proximity (minimizing diffusion-related limitations), spatial restriction (affecting processes requiring clearance) and alteration of the effects of the solvent. In a bulk solution, the dynamics of excited states are controlled not only by the electronic properties inherent to the molecule but also by the solvent properties (micropolarity and microviscosity). Inside the octa acid capsule, polarization effects will probably not play a major role because its interior mimics a benzene-like environment, providing a coherent framework for all encapsulated compounds [153]. The apparent absence of environmental reorganization contrasts with the behavior observed in the free solution. These attributes make the octa acid capsule an interesting tool for studying, in particular, the dynamics of excited states.

The last part of the review is divided into two sections. The first focuses on in silico studies of molecular encapsulation and its consequences. The second explores theoretical analyses of photochemical phenomena observed experimentally.

#### 2.4.1. Encapsulation of Guests

**Figure 18:**
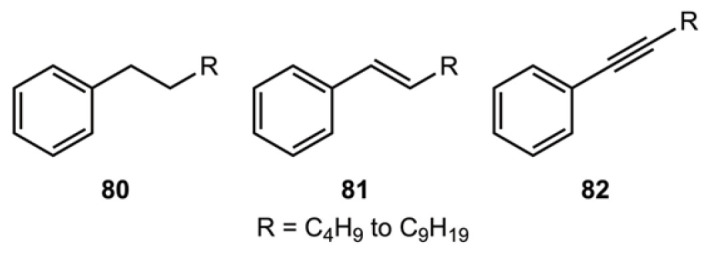
Guests studied in the octa acid capsule.

Ramamurthy and co-workers conducted a thorough study on the encapsulation of phenyl-substituted alkanes **80**, alkenes **81**, and alkynes **82** (Figure 18) within the octa acid capsule. They investigated the effects of length and the rigidity and presence of an unsaturation of the substituent [160]. The authors combined NMR experiments with molecular dynamics studies to investigate the conformational dynamics and encapsulation geometries of the host molecules.

Theoretical studies involved modeling and optimizing the guest molecules using the Chembio3D program with the MM2 force field [161]. Docking was performed using AutoDock Vina [162], and the most stable conformers were further refined with energy minimization in GROMACS, employing the OPLS-AA force field [163,164,165,166]. These methods provided a detailed view of the conformations of the guests within the capsule and their interactions with the host. In silico investigations revealed that molecular size played a critical role in the formation of capsuleplexes. Guests exceeding 15 Å in length were too large for encapsulation without folding their alkyl chains. For smaller molecules, linear conformations were favored, with the methyl group oriented near the narrower rim of the cavitand and the phenyl ring positioned closer to the aromatic moieties of the host. The only exception was dodecylbenzene (PhC_12_H_25_; length of 17.8 Å), where the phenyl ring, instead of residing in the aromatic cavity, was found in the broader middle region of the capsule, as observed by NMR spectroscopy. This positioning allows for possible π•••π interactions with the benzene rings of the host, facilitated by the molecule’s flexibility. In contrast, similar unsaturated guests lacked the necessary mobility for similar conformations, underscoring the role of molecular plasticity. Molecular dynamics simulations are key to understanding the dynamic processes by which guests achieve their stable conformations. Guests with short and unsaturated chains maintained their linear shapes throughout the 40 ns simulations. Molecules whose length is equal to or slightly exceeds the threshold of 15 Å, such as the *trans*-octylstyrene (PhCH=CHC_8_H_17_; length of 15.0 Å), change from linear to folded configurations after 10 ns and are stabilized in the central and wider region of the capsule. Heptylphenylacetylene (PhC≡CC_7_H_15_; length of 14.0 Å) exhibited rapid folding and unfolding during the initial nanoseconds before adopting a stable folded state, maximizing interactions with the inner walls of the capsule. Longer molecules, such as octylphenylacetylene (PhC≡CC_8_H_17_; length of 15.3 Å) and nonylphenylacetylene (PhC≡CC_9_H_19_; length of 16.4 Å), remained folded throughout their simulations. More flexible molecules like nonylbenzene (PhC_9_H_19_; length of 14.1 Å) demonstrated dynamic conformational behavior, occupying multiple positions within the capsule. In contrast, dodecylbenzene (PhC_12_H_25_; length of 17.8 Å) remained in the middle region.

It is well established that the *sine qua non* condition for forming an “octa acid” capsule is the presence of a guest. To better understand the formation of capsuleplexes, Choudhury and co-workers investigated the role of nonylbenzene (PhC_9_H_20_) in the self-assembly of the two cavitands **5** [167]. Previous theoretical studies revealed discrepancies between molecular docking predictions and experimental NMR observations [16]. Söderhjelm, Ryde and co-workers have noted that these deviations result from limitations in the docking approach, which neglects critical factors such as capsule and guest flexibility, conformational changes during complexation, and the involvement of solvent molecules and internal charges [168]. To simulate the capsuleplex formation, molecular dynamics studies were performed by positioning the macrocycles 10 Å apart, within the non-bonding interaction cut-off distance. Nonylbenzene was placed between the two cavitands, and the system was allowed to evolve and interact with its environment. The simulations spanned 500 ns and were conducted using the OPLS-AA force field implemented in the GROMACS 5.1.1 software at 300 K [163,164,165,166]. The initial event occurred within the first 10 ps, as the phenyl group of the guest approached the aromatic walls of one macrocycle. The alkyl chain adopted a folded loop conformation after 50 ps, facilitating the entry of the guest into the first cavity. Afterwards, the second macrocycle approached and interacted with the alkyl chain of the nonylbenzene, inducing a conformational change to an extended state. This process led to the formation of a partially open capsule after 50 ns, which rapidly evolved into a fully closed capsule by 55 ns. The resulting assembly remained stable throughout the simulation period, with only minor fluctuations. During encapsulation, the guest underwent significant folding and explored a range of conformations throughout the simulation. The final simulated capsuleplex aligned well with prior NMR observations. A control simulation conducted without the guest failed to produce a capsule, consistent with experimental findings [157].

#### 2.4.2. Studies of Excited States

**Figure 19:**
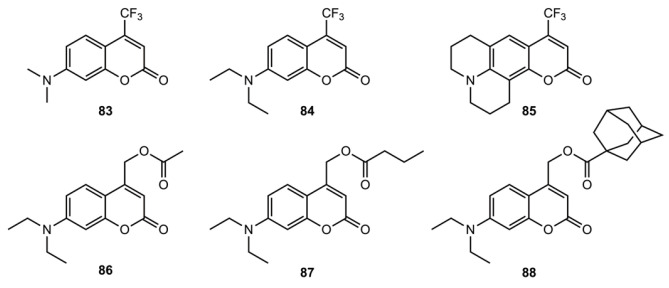
Courmarin derivatives studied inside the octa acid capsule.

Ramamurthy and co-workers have investigated the encapsulation behavior of coumarin derivatives **83**–**88** within the octa acid capsule (Figure 19) [169]. The study determined that the guest molecules remain isolated from the external aqueous solvent, a crucial factor for photochemical applications. Experimental and computational approaches were employed, with the Gaussian 09 program used to optimize the geometries of the organic capsule and the guest molecules at the B3LYP/6-31g(d) level [59,170]. Molecular docking was conducted using AutoDock Vina 1.5.6 to evaluate the binding affinities, while molecular dynamics simulations, performed with GROMACS 4.5.6 using the AMBER 03 force field, provided insights into the dynamic behavior of the capsuleplexes [162,171,172].

Molecular dynamics showed that the three fluorinated coumarin derivatives **83**–**85** were encapsulated in completely closed capsules. In contrast, derivatives **86**–**88** remained in contact with external water molecules. Specifically, derivatives **86** and **87** formed one and two hydrogen bonds with water, respectively, while the larger adamantyl-substituted derivative **88** interacted with at least six molecules of water. This trend is attributed to steric congestion, caused by the increasing steric hindrance of these guests, which induces partial capsule opening. Since coumarins exhibit solvent polarity-dependent emission maxima, quantum yields, and S_1_ lifetimes, the experimental results corroborate the computational findings regarding the presence or absence of a “dry” environment within the capsule. For derivatives **86**–**88**, known phototriggers, UV excitation triggered *α*-cleavage, resulting in two radical intermediates. One remained encapsulated, while the other, the carboxylic acid derivative, was released into the solution, further demonstrating this opening.

**Figure 20:**
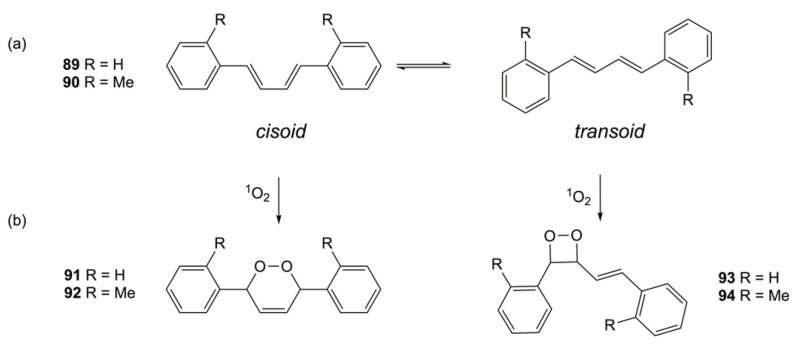
Studied 1,4-Diphenyl-1,3-butadiene (R = H) **89** and 1,4-ditolyl-1,3-butadiene (R = CH_3_) **90**; (**a**) *transoid*/*cisoid* isomer equilibrium; (**b**) reaction of each isomer with singlet oxygen.

The same group explored the formation of inclusion complexes with 1,4-diaryl-1,3-dienes and their reactivity toward the photoisomerization and photooxygenation of the guest [173]. The two dienes, namely 1,4-diphenyl-1,3-butadiene **89** and 1,4-ditolyl-1,3-butadiene **90**, adopt two conformations, *cisoid* and *transoid*, in equilibrium at room temperature (Figure 20a).

Experiments show that irradiating *transoid* dienes **89** and **90** and then reacting with singlet oxygen results exclusively in the formation of 6-membered ring endoperoxides **91** and **92** [174], rather than oxetanes **93** and **94** [175] (Figure 20b). The conversions for dienes **89** and **90** reach up to 79 and 57%, respectively, when the reaction is carried out in the presence of the cavitand **5**. Notably, a lower amount of endoperoxides **91** and **92** is formed in acetonitrile. Molecular dynamics simulations were carried out to understand this behavior, employing the OPLS-AA force field in GROMACS [163,164,165,176]. The guest molecules were optimized using Chembio3D and the MM2 force field and docked using the AutoDoc Vina program [161,162]. The computational analyses showed that all diene isomers preferentially adopted the *cisoid* conformation upon encapsulation. Solvent-accessible volume [177] calculations using the AMBER 03 force field [172] revealed that the *cisoid* form is more spherical and compact, making it a better fit for the cavity and enhancing van der Waals interactions. This preferential encapsulation explains the experimentally observed selectivity. The selective formation of the endoperoxides **91** and **92** confirms that only the *cisoid* form is present in the confined capsule environment, dictating the reaction pathway.

Elles and co-workers have studied the photoisomerization of stilbenes encapsulated in the octa acid capsule, focusing on how confinement affects their excited state dynamics [178]. The guests were encapsulated as monomers, and the experimental results showed significant changes in the *cis*/*trans* isomer distribution, excited state lifetimes, and quantum yields compared to their behavior in classical solvents [179,180]. In cyclohexane, the photostationary state of free 4,4′-dimethylstilbene **95** displayed a *trans*/*cis* ratio of 18/76 with 6% of phenanthrene, while within the octa acid capsule, this shifts dramatically to 80/20. Similarly, the photostationary state of 4-propylstilbene **96** changes from a *trans*/*cis* ratio of 20/80 in solution to 3/97 in the octa acid capsule. The alkyl substituents on the stilbene backbone have a marked effect on these ratios, highlighting the influence of substituent size and position. Molecular dynamics simulations were used to analyze the conformations and binding energies of the *trans* and *cis* isomers within the confined space of the octa acid capsule in order to understand these phenomena. The guests were optimized using B3LYP/6-31G* calculations via the Gaussian 09 software, while the dynamics were performed with the GROMACS 4.5.6 program using the AMBER03 force field for 100 ns with a 2-fs time step [59,170,171,172,181]. Binding energies were computed using the molecular mechanics/Poisson-Boltzmann surface area (MM/PBSA) method [182]. The in silico results revealed that the nature of the substituents on the stilbene moiety significantly influences the stability of encapsulated conformers. The position and size of the substituents modulate the interactions with the inner walls of the capsule. In solution, *trans* isomers generally exhibit greater stability. However, within the capsule, the *cis*/*trans* ratio was strongly dependent on the substituents. For unsubstituted stilbene **97**, the *trans* isomer is 1.9 kcal/mol more stable. For monosubstituted substrates, increasing alkyl chain length stabilizes the *cis* isomer, which becomes more favorable starting with 4-ethylstilbene **98** (by 2.4 kcal/mol) and increasing to 15.2 kcal/mol for 4-propylstilbene **96**. Among the disubstituted stilbenes, only 3,3′-dimethylstilbene **99** favored the *cis* isomer within the capsule. These computational results are in good agreement with experimental observations and provide an explanation for the altered photostationary states observed in the confined environment [180]. The experiments also revealed increased excited-state lifetimes within the octa acid capsule, particularly for 4-propylstilbene **96**, where the lifetime extension was most pronounced, up to 4.5 times longer than in cyclohexane. This is due to the substantial stabilization of the *cis* isomer relative to the *trans* isomer, driven by steric hindrance. This steric effect inhibits rotation around the central C=C bond, a critical step in isomerization that proceeds via torsional rotation [183]. In contrast, for 4,4′-dimethylstilbene **95**, the *trans* isomer is more stabilized within the capsule due to CH•••π interactions between the methyl groups and the inner aromatic walls of the octa acid cavity.

Gibb, Ramamurthy and co-workers reported an intriguing example of altered photochemical behavior within the capsule. They observed excimer formation of anthracene **42** in aqueous solution in the presence of octa acid capsule [184]. Under typical irradiation conditions, anthracene **42** typically photodimerizes [185,186]. However, in this encapsulated system, no photodimerization occurred. This deviation arises from the unique formation of capsuleplex, which hosts two molecules of anthracene **42**. The photodimerization product cannot be encapsulated due to its incompatibility with the cavity of the capsule. Instead, the capsule effectively prevents dimer formation while preassembling the system for excimer generation. Motivated by these observations, Srinivasan, Ramamurthy, Sen and co-workers conducted in silico studies to explore the stability of the two hosts in the capsule [187]. Molecular dynamics simulations, conducted using methodologies similar to those described in the previous example, revealed that the two anthracene **42** molecules are encapsulated in a slipped-sandwich arrangement. This configuration clearly facilitates π•••π stacking between the two anthracenes **42** and the aromatic walls of the capsule, while leaving no free space around the guests. This explains the suppression of photodimerization. The molecular dynamics simulations also suggested that the encapsulated arrangement is suboptimal for excimer formation, which is a key finding that will be further investigated through quantum chemical calculations. The authors used the QM/MM-TDDFT method with a 6-31g(d,p) basis set to map the excited-state surfaces [188,189]. The DFT-optimized ground-state structure closely matched the equilibrated structure obtained from molecular dynamics simulations. Upon excitation, different excimer configurations were identified and classified as anthracene-like, naphthalene-like, and benzene-like, based on their π•••π stacking arrangements [190]. Computational results indicate that within the capsule, the naphthalene-like excimer is the most stable form, contrasting with gas-phase conditions where the anthracene-like excimer dominates. This disparity arises from interactions with the capsule walls. Theoretical insights indicate that the anthracene-like excimer can stabilize if the capsule undergoes structural reorganization. This dynamic was not modeled due to computational limitations. Excitation to the S₁ state has been shown to induce guest motion, leading initially to the formation of the naphthalene-like excimer, followed by the anthracene-like excimer, and subsequent emission. Ultrafast spectroscopic measurements corroborated these computational findings, revealing multiple intermediates in the excited-state pathway.

**Figure 21:**
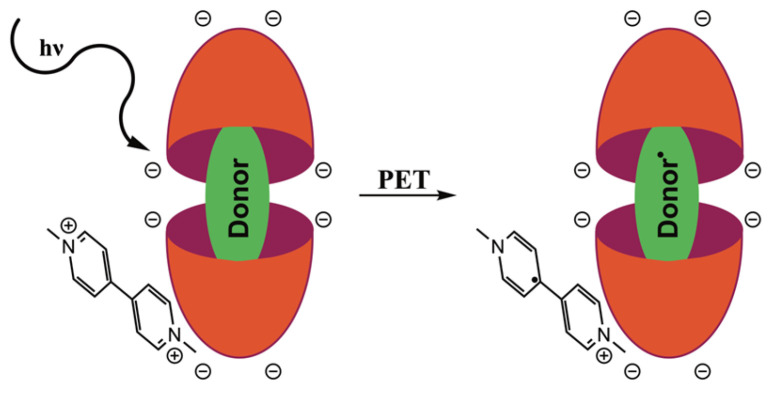
Dimethylviologen involved in PET with octa acid capsule.

Energy transfer mechanisms, Förster resonance energy transfer [191,192] and photoinduced electron transfer (PET) [193,194,195] are two other photophysical phenomena that have been rigorously studied in the supramolecular octa acid capsule [196]. Conventionally, PET necessitates direct interaction between an electron donor and an acceptor. However, this system has demonstrated the intriguing possibility of electron transfer through the rigid walls of the capsule, a notable deviation from classical PET paradigms. In the aforementioned studies, conducted by Ramamurthy and co-workers, electron transfers have been observed between various encapsulated donors, including coumarin or azulene derivatives, and viologen-based acceptors. Subsequent to the photoexcitation of the donors, an observation was made of a long-lived radical-ion pair, with a separation made by the capsular assembly. It is crucial to note that these transfers do not rely on the partial (~5 μs) or full disassembly of the host capsule. The disassembly of the capsule occurs on a second (~2.7 s) timescale [197,198]. Instead, PET occurs on a timescale faster than a nanosecond. This unequivocally rules out dynamic exchange or diffusion as the operative mechanism. This accelerated kinetics, in comparison to diffusion, underscores a static transfer mechanism, likely facilitated by proximity and the structural features of the capsule itself. In silico molecular dynamics simulations have been conducted, particularly involving coumarin **85** (Figure 19) and dimethylviologen **100** (Figure 21), using the OPLS-AA force field within the GROMACS software framework [163,164,165,166]. These simulations reveal that while the donor is securely encapsulated within the hydrophobic cavity, the acceptor is electrostatically tethered to the outer surface of the capsule. The positively charged moieties of the viologen interact with the anionic carboxylate groups of the capsule through Coulombic interactions. This results in the localization of the cationic species between the carboxylate groups at the extremities of the capsule and along its equator (Figure 21). The distance between the donor and acceptor was estimated to be 8.9 Å [194]. This model provides a compelling rationale for the absence of translational diffusion and is further supported by complementary NMR studies. In addition, TDDFT calculations within a polarizable continuum model (PCM), as reported by Ramamurthy, Dunietz and co-workers, provide significant insights into the excited-state behavior of coumarin derivatives [199]. Their findings indicate that the capsule itself can act as a transient electron acceptor from an excited donor, such as coumarin **85**. Subsequent to the transfer of the charge to the capsule wall, the electron is relayed to an external acceptor, such as a viologen species in close proximity. This observation underscores the active role of the capsule architecture, refuting the notion of it being merely a passive container and establishing it as a participant in the electron transfer chain.

These results underscore the multifaceted role of supramolecular capsules in mediating and directing charge transfer processes. The electron-conductive capability of the capsule walls themselves opens exciting new possibilities in the realms of biological electron transfer mimics and materials science, particularly in the design of nano-reactors and charge relay systems.

## 3. Conclusions

In silico studies have proven to be invaluable tools in supporting experimental hypotheses and providing new insights that are otherwise inaccessible. Supramolecular systems based on resorcinarenes have particularly benefited from advances in simulation methodologies, as highlighted in this review. This work has focused on hexameric assemblies of resorcinarenes and their pyrogallolarene homologues, as well as resorcinarene derivatives such as velcrands and octa acid-based systems.

One of the key contributions of in silico studies is their ability to provide information on the early stages of assembly formation, details that remain elusive to modern analytical techniques. In addition, these simulations allow the investigation of guest stoichiometry, guest conformation, and even mechanistic pathways of reactions occurring within confined cavities. Whenever possible, in silico results have been compared with experimental data, showing strong complementarities. For example, experimental calculations of product distributions from catalytic processes in confined systems have been successfully matched with computational predictions.

The continued improvement of in silico techniques holds great promise for the future of supramolecular chemistry. By enabling predictive modeling prior to experimental validation, these methods not only improve our understanding of confined systems but also pave the way for more efficient and innovative designs in supramolecular assembly and catalysis. These advances will undoubtedly contribute to the development of the next generation of confined systems with enhanced functionality and broader applicability.

## Figures and Tables

**Table 1:** Packing coefficients for each theoretical number of encapsulated guests with the effective number of encapsulations in bold.

Guest		Packing Coefficients Regarding Number of Guests						
	Volume (Å^3^)	in 3•3 Capsule (425 Å^3^)			in 3•56_4_•3 Capsule (620 Å^3^)			
		1	2	3	1	2	3	4
Ethane	46	11	22	33	7	15	22	30
Cyclopropane	56	13	26	**39**	9	18	27	**36**
*n*-Butane	80	19	**38**	57	13	26	**39**	52
*n*-Hexane	113	27	**53**	80	18	36	**54**	72

**Table 2:** Packing coefficients of guest for the different capsules with bold values for experimentally observed assemblies.

Guest			Supramolecular Assembly			
			3•57_4_•3	3•57_2_•57′_2_•3	3•57′_2_•57_4_•3	3•57_4_•57′_4_•3
Calculated Length (Å)			Accessible Cavity Length (Å)			
			19	20	24	28
	Extended	Coiled	Packing Coefficient (%)			
*n*-C_14_H_30_	19.2	14.4	**50**	47		
*n*-C_15_H_32_	20.5	15.3	**53**	50		
*n*-C_16_H_34_	21.7	16.2	55	**53**		
*n*-C_17_H_36_	23.0	17.2		**53**		
*n*-C_18_H_38_	24.3	18.1		**54**	52	
*n*-C_19_H_40_	25.5	19.1			**53**	
*n*-C_20_H_42_	26.8	20.1			**53**	
*n*-C_21_H_44_	28.0	21.0			55	**54**
*n*-C_22_H_46_	29.3	21.9				**55**
*n*-C_23_H_48_	30.6	22.7				**56**

**Table 3:** Energies of interaction for the dimers.

Dimer	Dimerization Energy (kcal/mol)	Interaction Energy with the Capsule (kcal/mol)	Total Interaction Energy with the Capsule (kcal/mol)
**79**•**79**-*trans*	15.4	38.7	56.3
**78**•**79**-*trans*	13.8	40.8	56.7
**78**•**78**-*trans*	11.5	41.7	55.3
**78**•**77**-*trans*	9.4	44.7	56.3
**77**•**79**-*trans*	7.6	44.3	57.2
**77**•**77**-*trans*	7.6	47.7	58.1
